# Cognitive and psychiatric symptom trajectories 2-3 years post-COVID-19 hospitalisation: a longitudinal prospective cohort study

**DOI:** 10.1016/S2215-0366(24)00214-1

**Published:** 2024-07-31

**Authors:** Maxime Taquet, Zuzanna Skorniewska, Thomas De Deyn, Adam Hampshire, William R. Trender, Peter J. Hellyer, James D. Chalmers, Ling-Pei Ho, Alex Horsley, Michael Marks, Krisnah Poinasamy, Betty Raman, Olivia C. Leavy, Matthew Richardson, Omer Elneima, Hamish J. C. McAuley, Aarti Shikotra, Amisha Singapuri, Marco Sereno, Ruth M. Saunders, Victoria C. Harris, Natalie Rogers, Linzy Houchen-Wolloff, Neil J. Greening, Parisa Mansoori, Ewen M. Harrison, Annemarie B. Docherty, Nazir I. Lone, Jennifer Quint, Christopher E. Brightling, Louise V. Wain, Rachael A. Evans, John R. Geddes, Paul J. Harrison

**Affiliations:** 1 Department of Psychiatry, University of Oxford, Oxford, UK; 2 Oxford Health NHS Foundation Trust, Oxford, UK; 3 NIHR Oxford Health Biomedical Research Centre, Oxford, UK; 4 Department of Neuroimaging, Institute of Psychiatry, Psychology and Neuroscience, King’s College London, UK; 5 Department of Brain Sciences, Imperial College London, United Kingdom; 6 University of Dundee, Ninewells Hospital and Medical School, Dundee, UK; 7 MRC Translational Immune Discovery Unit, University of Oxford, Oxford, UK; 8 Division of Infection, Immunity & Respiratory Medicine, Faculty of Biology, Medicine and Health, University of Manchester, Manchester, UK; 9 Manchester University NHS Foundation Trust, Manchester, UK; 10 Department of Clinical Research, London School of Hygiene & Tropical Medicine, London, UK; 11 Hospital for Tropical Diseases, University College London Hospital, London, UK; 12 Division of Infection and Immunity, University College London, London, UK; 13 Asthma and Lung UK, London, UK; 14 Radcliffe Department of Medicine, University of Oxford, Oxford, UK; 15 Oxford University Hospitals NHS Foundation Trust, Oxford, UK; 16 Department of Population Health Sciences, University of Leicester, Leicester, UK; 17 The Institute for Lung Health, Department of Respiratory Sciences, University of Leicester, Leicester, UK; 18 NIHR Leicester Biomedical Research Centre, University of Leicester, Leicester, UK; 19 University Hospitals of Leicester NHS Trust, Leicester, UK; 20 Therapy Department, University Hospitals of Leicester NHS Trust, Leicester, UK; 21 Long Covid Support, England and Wales, UK; 22 MQ: Transforming Mental Health, London, UK; 23 Centre for Medical Informatics, The Usher Institute, University of Edinburgh, Edinburgh, UK; 24 Usher Institute, University of Edinburgh, Edinburgh, UK; 25 Royal Infirmary of Edinburgh, NHS Lothian, Edinburgh, UK; 26 NHLI, Imperial College London, London, UK

## Abstract

**Background:**

COVID-19 is known to be associated with increased risks of cognitive and psychiatric outcomes. However, whether these symptoms can emerge or persist beyond the first year post-infection, what early aspects of the COVID-19 illness predict them, and how they relate to occupational functioning remain unknown. This study aimed to answer these questions.

**Methods:**

The Post-hospitalisation COVID-19 study (PHOSP-COVID) is a prospective, longitudinal cohort study of adults hospitalised with COVID-19 across the UK. Between 2 and 3 years post-hospitalisation, a subset of participants completed a computerised cognitive test, and clinical scales for subjective cognitive decline, depression, anxiety, and fatigue. We evaluated how the absolute risks of these symptoms evolved between the 6-month, 12-month, and 2-3-year follow-ups and whether symptoms at 2-3 years were predicted by earlier aspects of the COVID-19 illness. Additionally, we assessed whether participants changed their occupation, if so why, and which symptoms at 2-3 years were associated with occupational changes.

**Findings:**

475 participants provided data at the 2-3 years follow-up (mean [SD] age 58.3 [11.1] years, 40.2% female, 59.8% male). Participants had worse cognitive scores than would be expected for their sociodemographic characteristics, across all cognitive domains tested (average score 0.71 SD below the mean, IQR: 0.16–1.04 SD). Most participants reported at least mild depression, anxiety, fatigue, and subjective cognitive decline, and 22.4 – 24.9% reported severe depression, fatigue, and subjective cognitive decline. Depression, anxiety, and fatigue were worse at 2-3 years than at 6 and/or 12 months, with evidence of both worsening of existing symptoms and emergence of new symptoms. Symptoms at 2-3 years were not predicted by the severity of the acute COVID-19 illness but strongly predicted by the degree of recovery at 6 months (explaining 35.0-48.8% of the variance in anxiety, depression, fatigue, and subjective cognitive decline). A biocognitive profile linking acutely raised D-dimer relative to CRP with subjective cognitive deficits at 6 months also strongly predicted symptoms at 2-3 years, as did individual symptoms at 6 months. Objective cognitive deficits at 2-3 years were not predicted by any of the factors tested except for cognitive deficits at 6 months. Over 1 in 4 participants reported occupational change with poor health being the commonest reason for this. Occupation change was strongly and specifically associated with objective and subjective cognitive deficits at 2-3 years, rather than the other symptoms.

**Interpretation:**

Psychiatric and cognitive symptoms appear to increase over the first 2-3 years post-COVID-19 hospitalisation, due to both worsening of symptoms already present at 6 months, and emergence of new symptoms. New symptoms mostly occur in people with other symptoms already present at 6 months. Early identification and management of symptoms might therefore be an effective strategy to prevent later onset of a complex syndrome. Occupation change is common and mostly associated with objective and subjective cognitive deficits. Interventions to promote cognitive recovery or prevent cognitive decline are therefore needed to limit the functional and economic impacts of SARS-CoV-2 infections.

**Funding:**

National Institute for Health and Care Research Oxford Health Biomedical Research Centre, the Wolfson Foundation, MQ Mental Health Research, MRC-UK Research and Innovation, National Institute for Health and Care Research.

## Introduction

SARS-CoV-2 infection is associated with increased risks of neuropsychiatric disorders including depression, anxiety, and cognitive deficits,^1–5^ either in isolation or as part of a post COVID-19 syndrome (also known as long COVID).^6^ In studies based on electronic health records, these risks were found to be higher in individuals hospitalised with COVID-19.^1,3,7^ However, the lack of long-term prospective longitudinal data means that it is unknown if neuropsychiatric disorders emerge and/or persist beyond the first year post-infection, whether early aspects of COVID-19 illness predict later outcomes, and whether symptoms impact on occupational functioning.

Most studies investigating neuropsychiatric outcomes beyond 18 months post-infection relied on electronic health records.^2,3,7^ These cannot distinguish emergent disorders from delayed diagnosis and cannot ascertain the duration and severity of symptoms. Two prospective cohort studies with longer follow-ups investigated mental health outcomes post-COVID-19,^8,9^ including one that reported proportions of persistent symptoms.^9^ However, neither study determined the trajectories of emergent and persistent symptoms, nor did they assess cognitive deficits.

C-Fog is a Tier 3 PHOSP-COVID study in which a subgroup of the Post-HOSPitalisation COVID-19 cohort (PHOSP-COVID)^10,11^ was prospectively followed up for up to 3 years after their hospital admission. Here we report the results of this study, showing how cognitive, psychiatric, and fatigue symptoms emerged and evolved over time, which early aspects of the COVID-19 illness predict these outcomes, and how symptoms correlate with occupation change, thereby addressing one of the joint patients and clinicians’ key research questions.^12^

## Methods

### PHOSP-COVID study and timeline

We recruited participants from PHOSP-COVID, a large-scale long-term study of nearly 8,000 adults discharged from one of 83 participating UK National Health Service (NHS) hospitals with a clinical diagnosis of COVID-19 (between February 1, 2020 and March 31, 2021).^10,11^ A total of 2,469 participants consented to be recontacted for other research and were invited to complete a computerised cognitive test, clinical scales and an assessment of their occupation, that participants completed between November 23, 2022 and May 1, 2023 corresponding to a time since hospital admission of 21 to 38 months which we refer to as the 2-3 years follow-up. All participants were invited and no predetermined sample size was sought.

Details of the PHOSP-COVID study have been published elsewhere.^10,11,13^ Further details (including a STROBE diagram) are provided in the appendix pp. 11–13. Written informed consent was obtained from all study participants and electronic consent was provided for the 2-3 years follow-up. The study was approved by the Leeds West Research Ethics Committee (20/YH/0225) and is registered on the ISRCTN Registry (ISRCTN10980107). It follows the STROBE reporting guidelines.

### Cognitive, psychiatric, fatigue, and occupational assessment

At the 2-3 years follow-up, participants undertook eight computerised online tasks from the Cognitron battery (a platform assessing cognition remotely via web browsers),^14^ which differs from the Montreal Cognitive Assessment (MoCA) done at 6 and 12 months. The cognitive domains tested within Cognitron were immediate memory, simple reaction speed, two-dimensional mental manipulation, cognitive control, spatial working memory, spatial planning, verbal analogies, and delayed memory. Each task resulted in an accuracy-based score. Predefined quality control was applied to results.

Following cognitive testing, participants were invited to complete questionnaires: PHQ-9 for depression, GAD-7 for anxiety, occupation change (whether they work less than before COVID-19 and why), FACIT for fatigue and its impact on daily activities and function,^15^ and cognitive change index (CCI)-20 for subjective cognitive decline (modified to ask about change compared to before COVID-19).^16^ Predefined thresholds were applied to each scale to define mild, moderate, and severe symptom burden. More details are provided in the appendix pp. 13–18.

### Statistical analysis

Baseline characteristics were compared between respondents and all other participants of the PHOSP-COVID study. Characteristics with a standardised mean difference > 0.1 were considered different between the two groups. Using t-tests, outcomes at 2-3 years were compared between those who responded only after receiving a reminder and those who responded upon first invitation.

Cognitive scores were transformed to z-scores for each domain based on normative models (learned from the Great British Intelligence study^17^) accounting for age, sex, level of education, ethnicity, and whether English was the participant’s first language. Z-scores were averaged across cognitive domains to provide an overall cognitive score, indicating the number of standard deviations above/below the excepted score for the participant’s sociodemographic characteristics.

The evolution of outcomes measured at 6 months, 12 months, and 2-3 years were represented with alluvial diagrams. When the same instrument was used across time points, changes in outcomes between 6 months and 2-3 years and between 12 months and 2-3 years were assessed using paired t-tests. This was repeated among those with at least mild symptoms at both time points (to assess for worsening/improvement of existing symptoms) and among those with scores below the threshold of mild burden for at least one time point (to assess for emergence/remission of symptoms).

Five factors were assessed as possible predictors of fatigue, psychiatric and cognitive outcomes at 2-3 years using linear regressions adjusted for age, sex, and time since infection: (i) markers of acute severity including World Health Organization (WHO) clinical progression scale, National Early Warning Scores (NEWS) summarising physical observations, duration of hospitalisation, intensive care admission, pulmonary embolism, and delirium during admission, (ii) history of psychiatric/neurological comorbidity, and of myalgic encephalomyelitis (ME), chronic fatigue syndrome (CFS), fibromyalgia or chronic pain, (iii) recovery clusters defined in a previous study to represent the degree of impairment measured at 6 months post-COVID across different symptom domains^10^, (iv) clinical scales capturing each symptom domain at 6 months (adjusting for the same symptom domain as the outcome), and (v) two biocognitive profiles linking acute blood biomarkers and cognitive outcomes at 6 months.^18^ Benjamini & Hochberg correction for multiple testing was applied across outcomes.

We assessed which symptoms at 2-3 years were most associated with occupation change at the same time point using univariable logistic regressions and a multivariable logistic Lasso regression (to account for multicollinearity) including all clinical scales, the overall cognitive score, age, sex, and time since infection as independent variables. For each clinical scale found to be associated with occupation change, additional univariable and multivariable logistic Lasso regressions were computed with the items from that scale as independent variables. Adjusted risk ratios (RRs) were calculated using generalised linear models with binomial outcome and log link functions.

All analyses were conducted in R version 4.2.0 and used complete data at the 2-3 years follow-up with no imputation. Statistical significance was set at 2-sided p-values < 0.05. Further details about statistical analysis are provided in the appendix pp. 18-19.

### Role of the funding source

The funder of the study had no role in study design, data collection, data analysis, data interpretation, or writing of the manuscript.

## Results

A total of 475 participants (19.2% of those invited) provided data at the 2-3 years follow-up (mean [SD] age 58.3 [11.1] years, 40.2% female, 59.8% male; Table 1 and appendix p. 22). Compared to the rest of the PHOSP-COVID cohort, participants followed up at 2-3 years were more likely to be white, native English speakers, having a higher education level, higher income, and having better objective cognition but worse subjective cognition at 6 months. They were similar in terms of age, sex, pre-COVID comorbidities (except for a higher burden of diabetes and psychiatric/neurological conditions), and in terms of their depression, anxiety, and fatigue measured at 6 months. Compared to those who participated upon first invitation, those who required a reminder had significantly worse overall cognitive score at 2-3 years but similar depression, anxiety, fatigue, and subjective cognitive deficits (appendix p. 23).

**Fig. 1:**
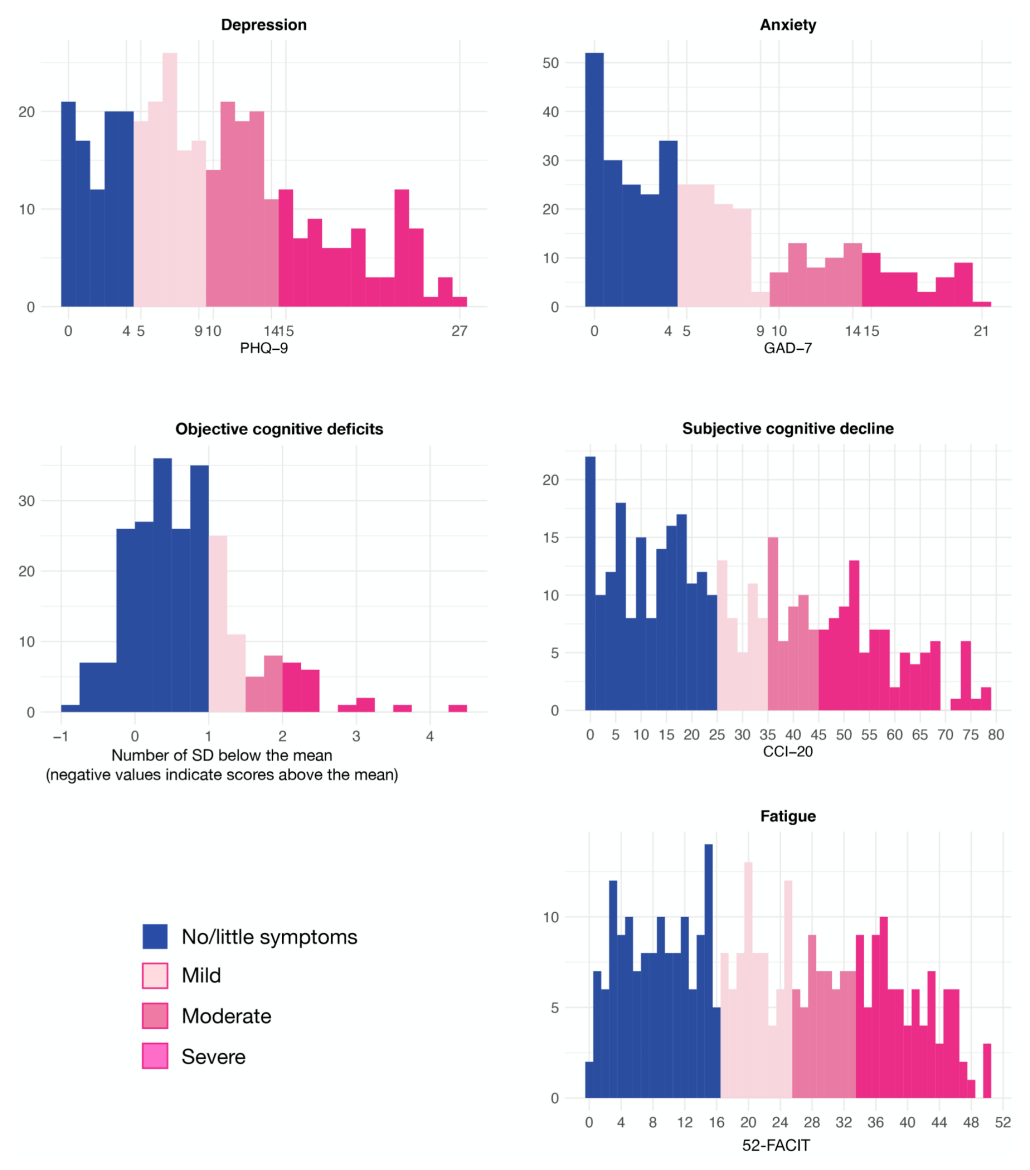
Distribution of the cognitive, psychiatric, and fatigue outcomes at 2-3 years post-COVID-19. The colours encode the severity based on predefined thresholds. For fatigue, the scale is inverted (reporting 52-FACIT) to match the interpretation that worse outcomes appear on the right.

**Fig. 2:**
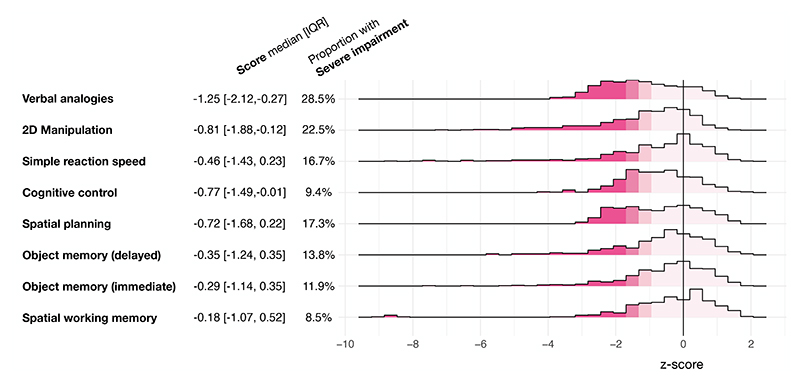
Distribution of the normalised scores for the different cognitive subdomains tested. The units represent the number of standard deviations below (negative) or above (positive) the mean for people with the same sociodemographic characteristics. For each domain, the mean score and its interquartile range (IQR) as well as the proportion of people with severe impairment (i.e. z-scores < -2) are provided. All distributions had mean significantly below zero (one-sample Wilcoxon test: p<0.0001).

Most participants reported at least mild depression (74.5%), anxiety (53.5%), fatigue (60.6%), and subjective cognitive decline (52.1%), with a substantial minority experiencing severe depression (22.4%), severe fatigue (24.6%), and severe subjective cognitive decline (24.9%; Figure 1). Participants had worse overall cognitive scores than would be expected for people of the same sociodemographic characteristics (but without COVID-19) by 0.71 SD (inter-quartile range [IQR] 0.16–1.04 SD, p<0.0001). Significant deficits were observed across all cognitive domains (Figure 2).

**Fig. 3:**
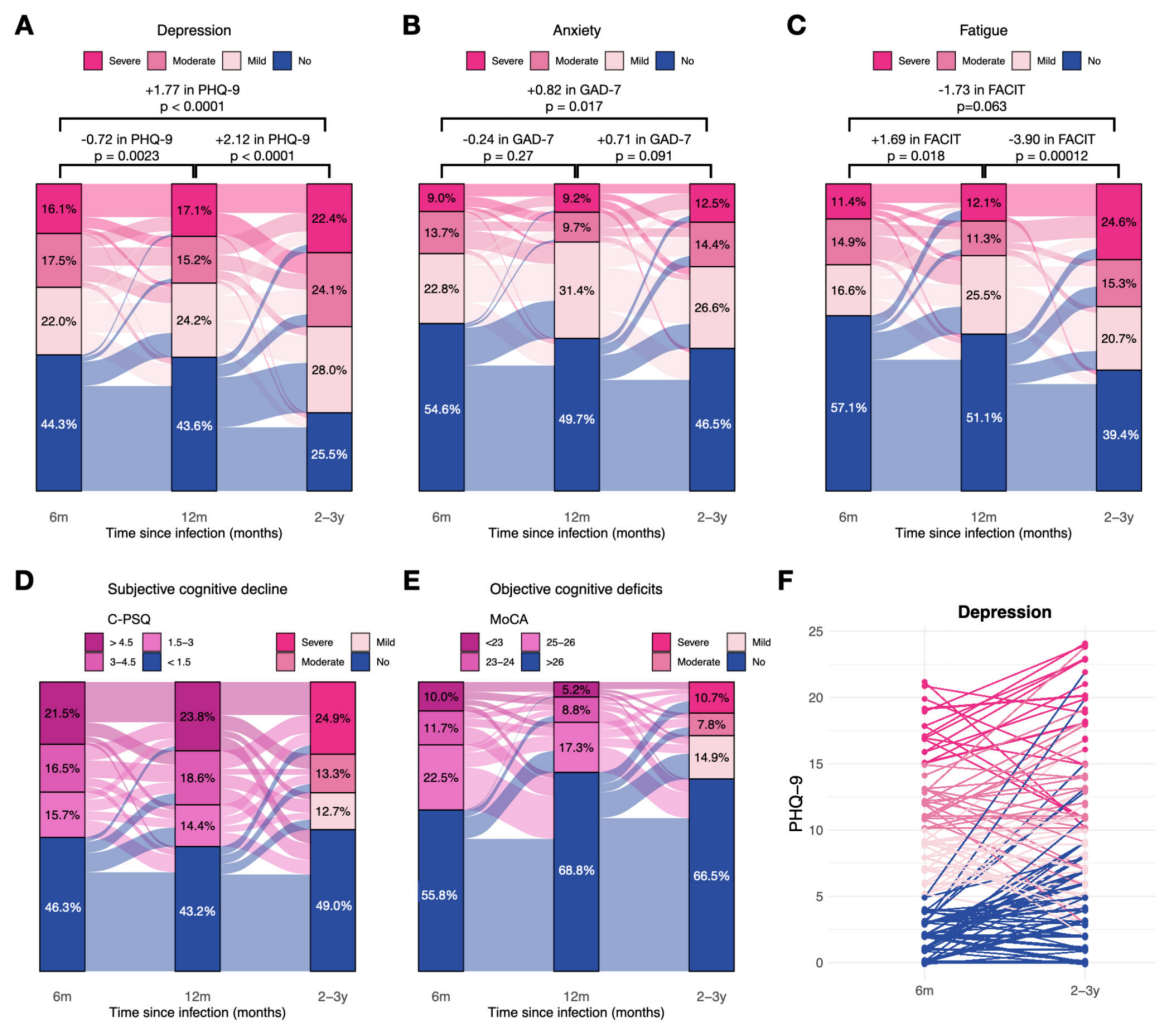
**A-E** Evolution of the proportion of participants with no, mild, moderate and severe burden of depression, anxiety, fatigue, and cognitive outcomes through follow-ups (among the same participants who provided data at different time points). For depression, anxiety, and fatigue, results of the paired t-tests are displayed in terms of the mean change in score and p-values (details, including confidence intervals can be found in the appendix p. 23). For fatigue, a negative change in FACIT means a worsening of symptoms, unlike for depression and anxiety. For objective and subjective cognitive outcomes, different scales were used at 2-3 years compared to 6 and 12 months and are therefore coloured differently. **F** Paired values of PHQ-9 at 6 months and 2-3 years. Graphs of paired values for GAD-7 and FACIT can be found in the appendix p. 20.

Evolution of the different scales from 6 months to 2-3 years based on data provided by the same individuals across time points are depicted in Figure 3 and appendix pp. 20 and 23. Depression increased from 6 months to 2-3 years. There was evidence of both worsening of persistent depressive symptoms (mean increase from 6 months to 2-3 years: 1.74, 0.50–2.99, p=0.0068) and a net emergence of new symptoms among people without symptoms at 6 months (mean increase 1.79, 0.68–2.91, p=0.0021). Anxiety also increased from 6 months to 2-3 years and there was evidence of net emergence of symptoms (increase in GAD-7 by 0.82, 0.058–1.58, p=0.035) while worsening of persistent symptoms was of similar magnitude but not statistically significant. Fatigue first improved from 6 to 12 months, before significantly deteriorating from 12 months to 2-3 years. Differences in fatigue scores between those with persistent symptoms and those with emerging/remitting symptoms were not significant. Incidences and remission proportions for all outcomes are presented in the appendix p. 24.

Among those with a recorded MoCA within normal range (> 26) at 6 and 12 months, 20.0% (95% CI 11.5–32.6%) had an overall cognitive score at 2-3 years at least 1 SD below the score expected for their sociodemographic characteristics. Among those who reported no subjective cognitive deficit at 6 and 12 months post-COVID-19 (as measured by the C-PSQ^18^), 7.7% (2.6–18.8%) reported at least some subjective cognitive decline at 2-3 years; and among those with subjective cognitive deficits at 6 and 12 months, 26.9% (17.7–38.6%) reported little to no decline at 2-3 years.

**Fig. 4:**
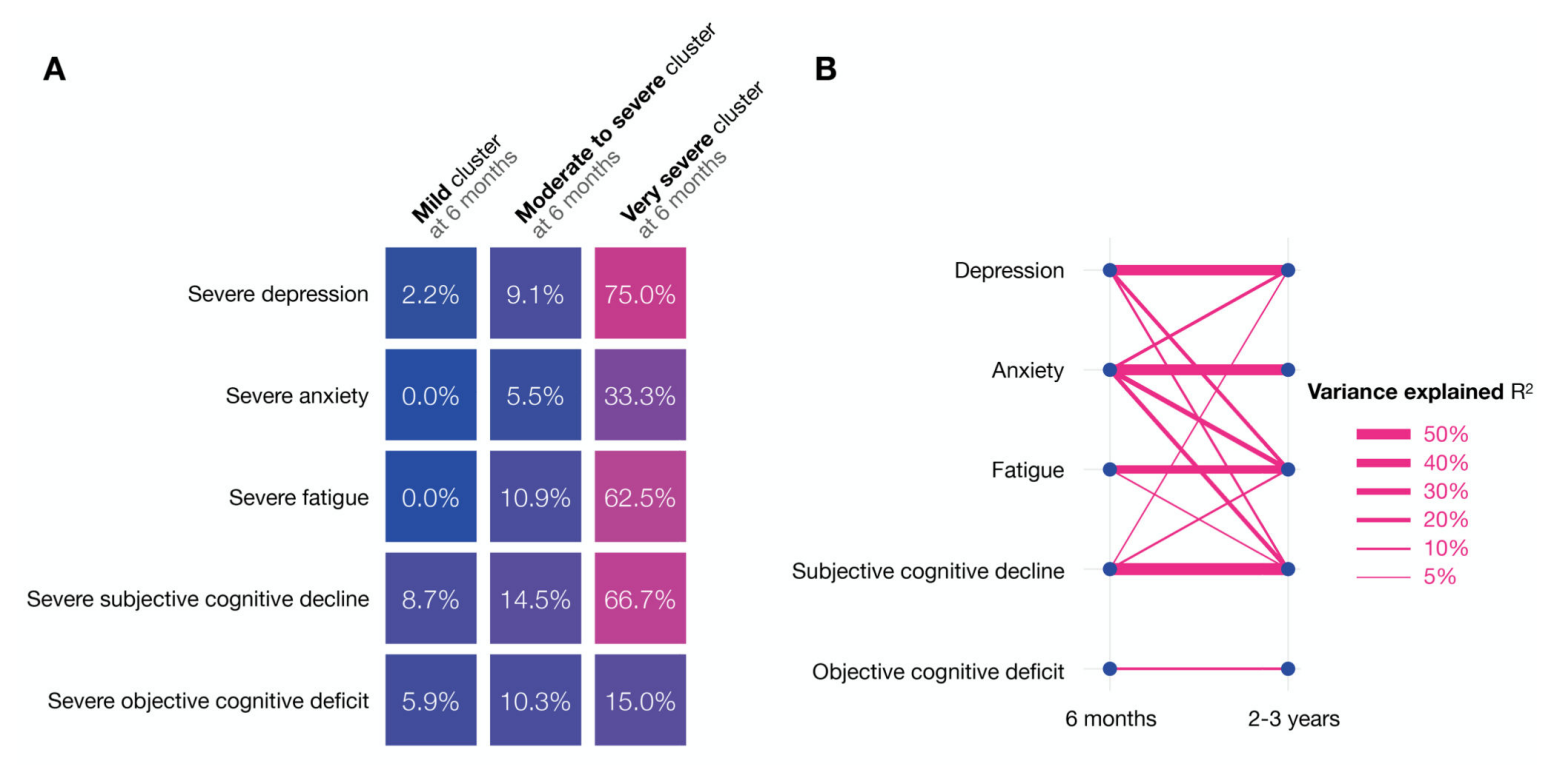
**A**. Prevalence of severe psychiatric, cognitive and fatigue outcomes at 2-3 years as a function of recovery at 6 months, based on three predefined clusters of recovery (one per column). **B** Prediction of symptom burden at 2-3 years based on symptoms at 6 months. Each line connecting symptom X at 6-months to symptom Y at 2-3 years represents the proportion of variance in Y at 2-3 years explained by symptom X at 6 months when adjusting for Y at 6 months. Only predictions that were significant at p<0.05 are represented. For subjective cognitive decline and objective cognitive deficits, the instrument used at 6 months and 2-3 years differ which might have led to a lower proportion of variance explained. All coefficients, p-values and R^2^ are provided in the appendix p. 26.

Severity of the acute illness did not predict outcomes at 2-3 years (Table 2). In contrast, the predefined clusters of recovery based on symptoms measured at 6 months^10^ strongly predicted symptoms, explaining 35-49% of the variance in depression, anxiety, fatigue, and subjective cognitive decline (Table 2). Those in the ‘very severe’ cluster of recovery at 6 months had substantial symptom burden at 2-3 years (Figure 4) including 75.0% experiencing severe depression, 66.7% reporting severe subjective cognitive deficits, 62.5% experiencing severe fatigue, 33.3% experiencing severe anxiety, and 15.0% having overall cognitive score 2 SD below the score expected for their sociodemographic characteristics. History of psychiatric or neurological condition increased the prevalence of most outcomes but history of ME/CFS, fibromyalgia, or chronic pain only increased the prevalence of fatigue and, to a lesser degree, depression. The biocognitive profile linking raised D-dimer relative to CRP during the acute illness with subjective cognitive deficits at 6 months^18^ significantly predicted most outcomes at 2-3 years, except for objective cognitive deficits (Table 2 and appendix p. 20). By contrast the biocognitive profile linking raised fibrinogen relative to CRP with both objective and subjective cognitive deficits at 6 months^18^ was not associated with any outcome at 2-3 years.

Over 1 in 4 participants reported having changed their occupation compared to before they had COVID-19 (95 out of 353, 26.9%, 95% CI 22.6–31.8%), and the main reason given was poor health (appendix p. 21). In univariable analyses, change in occupation at 2-3 years was found to be associated with subjective cognitive decline (adjusted odds ratio [OR] 1.54, 95% CI 1.21–1.98 and adjusted risk ratios [RR] 1.32, 1.14–1.56 for every point increase in CCI-20, p=0.00051), overall cognitive score (OR 1.51, 95% CI 1.04–2.22 and RR 1.34, 1.07–1.63 for every SD decrease in score, p=0.031), and fatigue (OR 1.31, 95% CI 1.03–1.69 and RR 1.22, 1.02–1.56 for every point decrease in FACIT, p=0.031). In sparse multivariable modelling, both overall cognitive score (OR 1.13) and subjective cognitive decline (OR 1.35) remained associated with change in occupation. The only two cognitive domains associated with occupation change were simple reaction speed (OR 1.34, 95% CI 1.16–1.55, p<0.0001 in univariable analysis, and OR=1.21 in sparse multivariable modelling) and cognitive control (OR 1.40, 95% CI 1.11–1.77, p=0.0047 in univariable analysis, and OR 1.27 in sparse multivariable modelling). All but one item of the subjective cognitive decline scale were significantly associated with occupation change in univariable analysis (appendix p. 27). Notably, in sparse multivariable modelling, the items selected to best correlate with occupation change were a worsening in ability to shift from one activity to the next (OR 1.13; univariable OR 1.61, 95% CI 1.26–2.05, p=0.00012), and a worsening in the ability to remember what one intended to do (OR 1.14; univariable OR 1.63, 95% CI 1.28–2.09, p=0.00010), whereas all other items had OR between 1.0 and 1.05 (appendix p. 27).

## Discussion

Individuals hospitalised with COVID-19 in the C-Fog cohort continue to experience substantial cognitive and psychiatric burden up to three years after infection. Almost 1 in 2 respondents to this study experienced moderate to severe depression, 1 in 4 reported severe cognitive decline, and 1 in 9 had objective signs of severe cognitive deficits (which would equate to a difference of 30 points on a typical IQ scale, in which 1 SD equals 15 points^14^). Fatigue added to this burden. Beyond symptoms, functional impact of COVID-19 hospitalisation was also evident: more than one in four participants reported a change in their occupation since having COVID-19. Depression, anxiety and fatigue increased from 6 months to 2-3 years. Symptoms at 2-3 years were best predicted by participants’ level of health impairment at 6 months and by a biocognitive profile linking raised D-dimer relative to CRP in the acute illness to 6-months subjective cognitive deficits.

Much of the burden can be attributed to persistence of symptoms already present 6 and 12 months post-COVID-19 hospitalisation (Figure 3). However, persistence alone cannot explain the significant increase in depression, anxiety, and fatigue scores from 6 months to 2-3 years post-COVID-19. The magnitude of the increase cannot be explained by aging of the cohort^15,19,20^ nor by the fact that the follow-up at 2-3 years was performed on a digital device rather than on paper.^21,22^ The increase seen may instead be explained by emergence of new symptoms or worsening of existing ones. For depression, there was robust evidence for both. For anxiety and fatigue, subgroup analyses were underpowered to tease apart the effects of worsening symptoms and newly emerging ones, but there was evidence of emerging anxiety symptoms. Overall, the findings regarding emerging symptoms are in keeping with the observed ongoing increased risk of new diagnoses of depression and anxiety beyond one year after COVID-19 hospitalisation.^7^ They support the hypothesis that this ongoing risk represents, at least in part, newly emergent symptoms and not just delayed diagnosis of persistent symptoms.

Emergence and worsening of cognitive deficits are more difficult to assess since different instruments were used to measure cognition at 6 and 12 months (MoCA for objective testing, and C-PSQ for subjective reporting) and at 2-3 years (Cognitron platform^14^ and CCI-20). The proportion of those with a normal MoCA at 6 and 12 months who had objective cognitive deficits at 2-3 years (20%) is greater than expected from the correspondence between MoCA and Cognitron scores.^23^ However, it might be that some participants with objective cognitive deficits at 6 and/or 12 months had a normal MoCA score because the MoCA is not sensitive to cognitive deficits in people with higher baseline cognition. As such, our data at 2-3 years provide a more accurate representation of the subsequent cognitive burden for people hospitalised with COVID-19. The ongoing cognitive burden at 2-3 years is compatible with the observation of ongoing increased risk of new diagnoses of cognitive deficits and dementia in those hospitalised with COVID-19.^7^ All cognitive domains were significantly affected, which mirrors results of a systematic review of smaller studies^24^ and a recent large cross-sectional study.^14^ Some participants had particularly low scores on specific tasks; these may reflect genuinely poor performance, misunderstanding of the task, or invalid responses not detected by the quality control. The lower participation of people with low MoCA scores at 6 months and the higher participation of those with cognitive deficits only after a reminder suggest that the reported cognitive deficits might underestimate the true burden. However, unmeasured confounding could also bias the estimate in the opposite direction.

Emergence of new symptoms need not be limited to people who were completely well 6 months after COVID-19. People who experienced symptoms in one domain (e.g. anxiety) might have started experiencing symptoms in another (e.g. depression). This is supported by the strong prediction of many symptom domains at 2-3 years by others at 6 months, even after adjusting for the other domain at 6 months (Figure 4B). Moreover, clusters of recovery at 6 months strongly predict all symptoms at 2-3 years. People in the ‘mild recovery’ cluster (including all those who were completely well at 6 months) experienced almost no severe symptoms at 2-3 years (Figure 4A). This contrasts with people in the ‘very severe’ cluster at 6 months – most of whom experienced severe depression, fatigue, and/or subjective cognitive decline at 2-3 years. It is therefore possible that from a single or a few symptoms emerges a network of symptoms (or syndrome). Such an emerging network between a range of post-acute features has been observed post-COVID more so than post-influenza.^6^ This network was found to become increasingly connected over time, possibly explaining the initial improvement (from 6 to 12 months) before a worsening in symptom burden. Whether such a symptom trajectory is specific to COVID-19 or is also observed in other illnesses remains to be determined in controlled studies. If a syndrome indeed emerges from a few core symptoms, then early interventions targeting the core symptoms might be a viable strategy to limit long-term symptom burden. In particular, anxiety at 6 months predicted many symptoms at 2-3 years. Identifying its aetiology and managing it early might reduce the symptom burden at 2-3 years. These hypotheses need testing in randomised controlled trials since the observational nature of this study make it prone to unmeasured confounding.

Beyond the symptom burden, assessing the impact of COVID-19 hospitalisation on occupation helps understand the functional consequences of COVID-19. The robust and specific association between occupation change and cognitive deficits (both objective and subjective) suggests that many people who changed occupation in the months and years after COVID-19 did so because they could no longer meet the cognitive demands of their job rather than for lack of motivation, interest, or confidence (which would all be reflected in an association with PHQ-9). Objective deficits in cognitive control, prolonged reaction time, and subjectively reported difficulties with switching activities and remembering what one intended to do were the best predictors of occupation changes. This suggests that people who changed occupation in the wake of COVID-19 have difficulties executing complex tasks with changing demands. Task switching is a particularly demanding cognitive process^25^ and important for performance in the workplace.^26^ Interventions such as brain training for task switching (provided it is acceptable to the patient and their fatigue level) might help reduce the impact of long COVID for the individual and the wider economy.^27^

A study of this kind cannot identify the mechanisms underpinning the different symptom trajectories, but it can provide some clues. No association was found between symptom burden and a range of markers of severity of the acute illness suggesting that the latter cannot explain the psychiatric and cognitive burden (amongst those whose illness severity had required hospitalization). In a previous analysis of the PHOSP-COVID study, two biocognitive profiles were found to link acute blood biomarkers with cognitive deficits 6 and 12 months post-COVID-19.^18^ Here we found that the profile linking raised D-dimer relative to CRP with subjective cognitive deficits at 6 months explains about 10% or more of the variance in depression, fatigue, and subjective cognitive decline at 2-3 years. This supports the hypothesis that this biocognitive profile captures a biological process with enduring consequences such as microthrombi in the cerebral vasculature.^18^ Conversely, the biocognitive profile linking raised fibrinogen relative to CRP with objective and subjective cognitive deficits was no longer associated with any symptoms at 2-3 years post-COVID, suggesting that it corresponds to a transient biological process such as neuroinflammation.^28^ These biological explanations remain hypotheses that require testing in mechanistic studies.

Objective cognitive deficits stood out as an outcome: they were not predicted by any of the other symptoms (not even subjective cognitive deficits) nor did they predict any other symptoms (Figure 4), and unlike other symptoms, they were not predicted by biocognitive profiles, history of neurological/psychiatric comorbidity, or clusters of recovery (Table 2). This suggests that objective cognitive deficits might have their own separate neurobiology, whereas mechanisms underpinning subjective cognitive decline might in part be shared with fatigue, depression, and anxiety.

This study has several strengths including a longitudinal follow-up for up to 3 years, detailed phenotyping of cognitive and psychiatric symptoms using validated instruments, and the assessment of both clinical and occupational impacts. It also has limitations. First, data are limited to patients hospitalised with COVID-19 and might not generalise to patients who were not hospitalised. In addition, the low response proportion (19.2%) means there is a risk of selection bias. Comparison at baseline and at 6-month follow-up can help assess how respondents differed from non-respondents: the two groups were similar in many baseline characteristics and 6-month outcomes but differed in other aspects (e.g. more likely to have higher education level and higher cognitive score at 6 months). Differences between those who required a reminder to participate and those who did not provide additional clues about differences between respondents and non-respondents (assuming the latter are more like those who required a reminder). The fact that they only differed in objective cognitive deficits provides evidence against large discrepancies between respondents and non-respondents. However, besides these measured characteristics, there might also be unmeasured differences between respondents and non-respondents that affect outcomes at 2-3 years post-COVID. Results (especially absolute risks) should therefore be interpreted cautiously. Second, because of the focus on a long follow-up, participants were all diagnosed early in the pandemic (before emergence of the delta variant) and results might not apply to people infected with other variants and people who were vaccinated prior to being infected. Although variants have changed the risks of cognitive and psychiatric outcomes,^2^ prior vaccination is not associated with a lower risk of psychiatric outcomes.^5,29,30^ Third, we do not know which participants have been reinfected nor their vaccination status after they had COVID-19. While reinfection^8^ and subsequent vaccination^31^ might affect absolute risks, they are likely to affect the cohort as a whole so that contrasts between subgroups remain similar. Fourth, the absence of a control group of individuals who never had COVID-19 means that it is unclear whether psychiatric and cognitive outcomes would have been observed during the study period in this population in the absence of COVID-19. However, the increased risk of cognitive and psychiatric diagnoses within 2 years post-COVID-19 hospitalisation compared to hospitalisation for other causes^7^ or the general population^3,4^ is well established and this study focused on identifying symptom trajectories and their predictors.

In summary, psychiatric and cognitive symptoms continue to be present up to three years after infection in a significant proportion of people hospitalised for COVID-19, and fatigue adds to this burden. The burden increased from 6 months to 2-3 years likely due to both a worsening of existing symptoms and the onset of new ones. Newly arising symptoms mostly affect people with symptoms in other domains at 6 months, which might reflect the emergence of a syndrome stemming from an individual symptom. As such, early treatment of the initial symptom domain might be an effective way of preventing later onset of a complex syndrome. Adults with severe ongoing health impairments at 6 months are at particularly high risk of severe symptoms at 2-3 years. Medical attention and follow up are warranted for this group. Occupation change is a common outcome in people hospitalised with COVID-19, especially those with objective and subjective cognitive deficits. Interventions to promote cognitive recovery and/or prevent cognitive decline are therefore needed to limit the functional and economic impacts of COVID-19 infections.

## Supplementary Material

Appendix

## Figures and Tables

**Table 1:** Baseline characteristics for the cohort of participants who reported data at 2-3 years compared to all other participants in the PHOSP-COVID cohort. SMD = Standardised mean differences.

	Cohort	Others	SMD
**Number**	475	7460	-
**SOCIODEMOGRAPHICS**			
Age, years; mean (SD)	58.26 (11.13)	59.32 (13.53)	0.079
Sex; n (%)			
Female	191 (40.21)	3015 (40.46)	0.0052
Male	284 (59.79)	4436 (59.54)	0.0052
Race; n (%)			
Asian	25 (5.26)	661 (8.88)	0.14
Black	12 (2.53)	363 (4.87)	0.12
Mixed	<10 (-)	114 (1.53)	-
White	417 (87.79)	5881 (78.97)	0.24
Other	15 (3.16)	428 (5.75)	0.13
Education; n (%)			
None	<10 (-)	157 (2.37)	-
Primary school	<10 (-)	168 (2.54)	-
Secondary school	113 (25.17)	2148 (32.47)	0.16
Sixth form college	62 (13.81)	816 (12.33)	0.044
Vocational qualification	66 (14.70)	771 (11.65)	0.09
Undergraduate university degree	80 (17.82)	916 (13.85)	0.11
Post-graduate qualification	89 (19.82)	725 (10.96)	0.25
Prefer not to say	29 (6.46)	915 (13.83)	0.25
Income; n (%)			
< £19,000	52 (14.40)	1122 (27.45)	0.32
£19,001- £26,000	61 (16.90)	696 (17.03)	0.0034
£26,001 - £35,000	46 (12.74)	605 (14.80)	0.06
£35,001 - £48,000	73 (20.22)	580 (14.19)	0.16
> £48,001	129 (35.73)	1085 (26.54)	0.20
English as first language; n (%)	415 (93.05)	5517 (81.08)	0.36
**COMORBIDITIES**, n (%)			
Cardiovascular condition	213 (45.22)	3667 (49.36)	0.083
Cerebrovascular accident	<10 (-)	296 (3.99)	-
Psychiatric or neurological condition	115 (24.47)	1433 (19.29)	0.13
ME/CFS/Fibromyalgia/Chronic pain	26 (5.52)	314 (4.22)	0.06
Diabetes	81 (17.09)	1681 (22.60)	0.14
Respiratory condition	158 (33.33)	2254 (30.30)	0.065
Rheumatological condition	82 (17.30)	1272 (17.09)	0.0057
Gastrointestinal condition	104 (22.03)	1472 (19.82)	0.055
Endocrine condition	41 (8.70)	686 (9.24)	0.019
Chronic kidney disease	16 (3.38)	416 (5.59)	0.11
Cancer	29 (6.13)	579 (7.79)	0.065
Chronic infection	10 (2.11)	185 (2.50)	0.026
**Clinical features at 6 months**, mean (SD)			
Objective cognitive function (MoCA)	26.89 (2.42)	25.54 (3.60)	0.38
Subjective cognitive function (C-PSQ)	2.53 (2.15)	2.05 (2.05)	0.23
Depression (PHQ-9)	6.99 (6.05)	7.05 (6.60)	0.0084
Anxiety (GAD-7)	4.91 (5.12)	5.38 (5.75)	0.082
Fatigue (inverse FACIT)	17.91 (12.46)	17.88 (13.38)	0.002
**Clusters of recovery at 6 months**, n (%)			
Mild	57 (34.55)	666 (29.73)	0.10
Moderate to Severe	76 (46.06)	1103 (49.24)	0.064
Very Severe	32 (19.39)	471 (21.03)	0.041

**Table 2:** Prediction of outcomes by factors representing different earlier aspects of the participant’s illness. Each cell in the table contains the proportion of variance explained (in %) by the predictor in a model first adjusted for age, sex, and time since infection. The p-values are Benjamini & Hochberg-corrected for each predictor independently. Bold cells highlight statistically significant results. WHO=World Health Organization Clinical Progression Scale, NEWS = National Early Warning Score, PE=Pulmonary embolism, ICU=Intensive Care Unit. All coefficients and unadjusted p-values can be found in the appendix p. 25.

	Depression	Anxiety	Fatigue	Subjective cognitive decline	Objective cognitive deficit
**WHO**	0.83 (p=0.66)	2.31 (p=0.24)	0.36 (p=0.75)	1.02 (p=0.66)	0.99 (p=0.66)
**NEWS**	0.044 (p=0.94)	0.017 (p=0.94)	0.016 (p=0.94)	0.0019 (p=0.94)	0.20 (p=0.94)
**Duration of admission**	0.015 (p=0.91)	0.52 (p=0.76)	0.31 (p=0.76)	0.0035 (p=0.91)	0.12 (p=0.91)
**ICU admission**	0.41 (p=0.62)	0.95 (p=0.62)	0.36 (p=0.62)	1.49 (p=0.62)	0.14 (p=0.72)
**PE**	0.28 (p=0.86)	1.15 (p=0.47)	0.14 (p=0.86)	1.86 (p=0.32)	3.27 (p=0.16)
**Delirium**	0.29 (p=0.97)	0.52 (p=0.97)	0.00056 (p=0.97)	0.029 (p=0.97)	0.22 (p=0.97)
**History of psychiatric/neurological comorbidity**	10.87 (p<0.0001)	6.44 (p<0.0001)	8.27 (p<0.0001)	7.19 (p<0.0001)	0.13 (p=0.59)
**History of ME/CFS/Fibromyalgia/Chronic pain**	1.62 (p=0.044)	0.99 (p=0.11)	3.53 (p=0.0022)	0.86 (p=0.11)	0.49 (p=0.29)
**Recovery cluster**	48.84 (p<0.0001)	39.43 (p<0.0001)	47.46 (p<0.0001)	35.04 (p<0.0001)	2.93 (p=0.12)
**Biocognitive profile (D-dimer)**	11.04 (p=0.0016)	7.02 (p=0.0089)	17.16 (p=7.5e-05)	9.75 (p=0.0023)	0.004 (p=0.96)
**Biocognitive profile (Fibrinogen)**	0.46 (p=0.62)	4.87 (p=0.13)	0.82 (p=0.61)	2.53 (p=0.28)	0.21 (p=0.70)

## Data Availability

The protocol, consent form, definition and derivation of clinical characteristics and outcomes, training materials, regulatory documents, requests for data access and other relevant study materials are available online at https://www.phosp.org.

## Research in context

### Evidence before this study

We searched PubMed (search updated on April 25, 2024) for papers published from March 22, 2022 (because we were interested in follow-up of at least 2 years and because a previous review had ended with a search on March 21, 2022 with no article directly relevant identified) until the search date with the terms (neuropsychiatr*[Title/Abstract] OR neurologic*[Title/Abstract] OR psychiatric[Title/Abstract] OR depress*[Title/Abstract] OR anxiety*[Title/Abstract] OR cognit*[Title/Abstract] OR brain[Title/Abstract]) AND (evolution[Title/Abstract] OR longitudinal[Title/Abstract] OR trajector*[Title/Abstract]) AND (COVID*[Title] OR SARS*[Title] OR coronavirus[Title]). We found several studies based on electronic health records data and several studies with follow-ups up to 18 months. We found one prospective cohort study with follow-up up to 3 years which did not assess cognition nor whether symptoms were emergent or persistent; one study of 51 patients followed up for 2 years but without statistical analyses; and one prospective study investigating which baseline characteristics are associated with psychiatric symptoms at 2 years (but without assessment of cognition). None of the studies with follow-up beyond 18 months investigated occupational impact.

### Added value of this study

To our knowledge, this is the first prospective cohort study which assesses trajectories of psychiatric and cognitive symptoms over the first 2-3 years after hospitalisation for COVID-19. We found that the burden of symptoms increased compared to 6 and 12 months post-COVID due to both worsening of existing symptoms and emergence of new ones. We also found that emergence of new symptoms mostly occurred in people with other symptoms present at 6 and 12 months, rather than people who were completely well at those earlier time points. A significant minority of people changed their occupation at 2-3 years post-hospitalisation compared to before they had COVID-19 and were working part time or not working at all, with the most common reason given being poor health. Occupation change was strongly and specifically associated with subjective cognitive decline and objective cognitive deficits rather than with anxiety, depression or fatigue.

### Implications of all the available evidence

The neuropsychiatric symptom burden among people who were hospitalised with COVID-19 has not disappeared 2-3 years post-infection and appears to have increased compared to 6 and 12 months post-infection. Prompt interventions to treat symptoms present in the months after hospitalisation might prevent the emergence of additional symptoms and the development of a more complex syndrome. Interventions promoting cognitive recovery or preventing cognitive decline might limit the occupational impact of SARS-CoV-2 infections thereby improving the functional and economic outcomes of COVID-19 for the individual and society at large.

## References

[1] Taquet M, Geddes JR, Husain M, Luciano S, Harrison PJ (2021). 6-month neurological and psychiatric outcomes in 236 379 survivors of COVID-19: a retrospective cohort study using electronic health records. Lancet Psychiatry.

[2] Taquet M (2022). Neurological and psychiatric risk trajectories after SARS-CoV-2 infection: an analysis of 2-year retrospective cohort studies including 1 284 437 patients. Lancet Psychiatry.

[3] Bowe B, Xie Y, Al-Aly Z (2023). Postacute sequelae of COVID-19 at 2 years. Nat Med.

[4] Xie Y, Xu E, Al-Aly Z (2022). Risks of mental health outcomes in people with covid-19: cohort study. BMJ.

[5] Wang Y, Su B, Xie J, Garcia-Rizo C, Prieto-Alhambra D (2024). Long-term risk of psychiatric disorder and psychotropic prescription after SARS-CoV-2 infection among UK general population. Nature Human Behaviour.

[6] Taquet M (2021). Incidence, co-occurrence, and evolution of long-COVID features: A 6-month retrospective cohort study of 273,618 survivors of COVID-19. PLoS Med.

[7] Ley H, Skorniewska Z, Harrison PJ, Taquet M (2023). Risks of neurological and psychiatric sequelae 2 years after hospitalisation or intensive care admission with COVID-19 compared to admissions for other causes. Brain Behav Immun.

[8] Zhang H (2023). 3-year outcomes of discharged survivors of COVID-19 following the SARS-CoV-2 omicron (B.1.1.529) wave in 2022 in China: a longitudinal cohort study. Lancet Respir Med.

[9] Guillen-Burgos HF (2023). Factors associated with mental health outcomes after COVID-19: A 24-month follow-up longitudinal study. Gen Hosp Psychiatry.

[10] Evans RA (2021). Physical, cognitive, and mental health impacts of COVID-19 after hospitalisation (PHOSP-COVID): a UK multicentre, prospective cohort study. Lancet Respir Med.

[11] Cohort Profile: Post-Hospitalisation COVID-19 (PHOSP-COVID) study. International Journal of Epidemiology.

[12] Houchen-Wolloff L (2022). Joint patient and clinician priority setting to identify 10 key research questions regarding the long-term sequelae of COVID-19. Thorax.

[13] PHOSP-COVID Collaborative Group (2022). Clinical characteristics with inflammation profiling of long COVID and association with 1-year recovery following hospitalisation in the UK: a prospective observational study. Lancet Respir Med.

[14] Hampshire A (2024). Cognition and Memory after Covid-19 in a Large Community Sample. New England Journal of Medicine.

[15] Montan I, Löwe B, Cella D, Mehnert A, Hinz A (2018). General population norms for the Functional Assessment of Chronic Illness Therapy (FACIT)-Fatigue Scale. Value Health.

[16] Rattanabannakit C (2016). The Cognitive Change Index as a measure of self and informant perception of cognitive decline: Relation to neuropsychological tests. J Alzheimers Dis.

[17] Hampshire A (2021). Cognitive deficits in people who have recovered from COVID-19. EClinicalMedicine.

[18] Taquet M (2023). Acute blood biomarker profiles predict cognitive deficits 6 and 12 months after COVID-19 hospitalization. Nat Med.

[19] Kocalevent R-D, Hinz A, Brähler E (2013). Standardization of the depression screener patient health questionnaire (PHQ-9) in the general population. Gen Hosp Psychiatry.

[20] Hinz A (2017). Psychometric evaluation of the Generalized Anxiety Disorder Screener GAD-7, based on a large German general population sample. J Affect Disord.

[21] Erbe D, Eichert H-C, Rietz C, Ebert D (2016). Interformat reliability of the patient health questionnaire: Validation of the computerized version of the PHQ-9. Internet Interv.

[22] Hirao K (2023). Differences in Center for Epidemiologic Studies Depression Scale, Generalized Anxiety Disorder-7 and Kessler Screening Scale for Psychological Distress scores between smartphone version versus paper version administration: Evidence of equivalence. Int J Environ Res Public Health.

[23] Bălăeţ M (2024). Online cognitive monitoring technology for people with Parkinson’s disease and REM sleep behavioural disorder. NPJ Digit Med.

[24] Fanshawe JB (2024). Cognitive domains affected post-COVID-19; a systematic review and meta-analysis. European Journal of Neurology.

[25] Monsell S (2003). Task switching. Trends Cogn Sci.

[26] Leroy S (2009). Why is it so hard to do my work? The challenge of attention residue when switching between work tasks. Organ Behav Hum Decis Process.

[27] Owen AM (2010). Putting brain training to the test. Nature.

[28] Petersen MA, Ryu JK, Akassoglou K (2018). Fibrinogen in neurological diseases: mechanisms, imaging and therapeutics. Nat Rev Neurosci.

[29] Al-Aly Z, Bowe B, Xie Y (2022). Long COVID after breakthrough SARS-CoV-2 infection. Nat Med.

[30] Taquet M, Dercon Q, Harrison PJ (2022). Six-month sequelae of post-vaccination SARS-CoV-2 infection: A retrospective cohort study of 10,024 breakthrough infections. Brain Behav Immun.

[31] Tran V-T, Perrodeau E, Saldanha J, Pane I, Ravaud P (2023). Efficacy of first dose of covid-19 vaccine versus no vaccination on symptoms of patients with long covid: target trial emulation based on ComPaRe e-cohort. BMJ Med.

