# Supplementary material for: Cognitive and psychiatric symptom trajectories 2-3 years post-COVID-19 hospitalisation: a longitudinal prospective cohort study
Source: Lancet Psychiatry. Author manuscript; Available in PMC 2026 Mar 14. (PMC7618856; doi:10.1016/S2215-0366(24)00214-1)
Supplement: Appendix [file EMS212625-supplement-Appendix.pdf]

PHOSP-COVID Collaborative Group

Core Management Group

Chief Investigator C E Brightling, *Members* R A Evans (Lead Co-I), L V Wain (Lead Co-I), J D Chalmers, V C Harris, L P Ho, A Horsley, M Marks, K Poinasamy, B Raman, A Shikotra, A Singapuri

PHOSP-COVID Study Central Coordinating Team

C E Brightling (Chief Investigator), R A Evans (*Lead Co-I*), L V Wain (*Lead Co-I*), R Dowling, C Edwardson, O Elneima, S Finney, N J Greening, B Hargadon, V C Harris, L Houchen--Wolloff, O C Leavy, H J C McAuley, C Overton, T Plekhanova, R M Saunders, M Sereno, A Singapuri, A Shikotra, C Taylor, S Terry, C Tong, B Zhao

Steering Committee

Co-chairs D Lomas, E Sapey*, Institution representatives* C Berry, C E Bolton, N Brunskill, E R Chilvers, R Djukanovic, Y Ellis, D Forton, N French, J George, N A Hanley, N Hart, L McGarvey, N Maskell, H McShane, M Parkes, D Peckham, P Pfeffer, A Sayer, A Sheikh, A A R Thompson, N Williams and core management group representation

Executive Board

Chair C E Brightling, representation from the core management group, each working group and platforms

Platforms

Bioresource

W Greenhalf (*Co-Lead*), M G Semple (*Co-Lead*), M Ashworth, H E Hardwick, L Lavelle-Langham, W Reynolds, M Sereno, R M Saunders, A Singapuri, V Shaw, A Shikotra, B Venson, L V Wain

Data Hub

A B Docherty (*Co-Lead*), E M Harrison (*Co-Lead*), A Sheikh (*Co-Lead*), J K Baillie, C E Brightling, L Daines, C Efstathiou, R A Evans, R Free, L Gardiner, A F Goemans, B Guillen-Guio, S Kerr, O C Leavy, F Liew, N I Lone, D Lozano-Rojas, H J C McAuley, K Ntotsis, R Pius, J Quint, M Richardson, , M Sereno, I D Stewart, M Taquet, M Thorpe, L V Wain

Genetic Analysis

L V Wain (Co-Lead), J K Baillie (*Co-Lead*), N Avramidis, E Coughlan, B Guillen-Guio, O C Leavy, E Pairo-Castineira, K Rawlik

Imaging Alliance

M Halling-Brown (*Co-Lead*), F Gleeson (*Co-Lead*), J Jacob (*Co-Lead*), S Neubauer (*Co-Lead*) B Raman (*Co-Lead*) S Siddiqui (*Co-Lead*) J M Wild (*Co-Lead*), S Aslani, G Baxter, M Beggs, C Bloomfield, M P Cassar, A Chiribiri, E Cox, D J Cuthbertson, M Halling-Brown, V M Ferreira, L Finnigan, S Francis, P Jezzard, G J Kemp,

H Lamlum, E Lukaschuk, C Manisty, G P McCann , C McCracken, K McGlynn , R Menke , C A Miller , A J Moss, T E Nichols, C Nikolaidou , C O'Brien , G Ogbole, B Rangelov, D P O'Regan , A Pakzad, S Piechnik , S Plein, I Propescu, A A Samat, L Saunders, Z B Sanders, R Steeds, T Treibel, E M Tunnicliffe, M Webster, J Willoughby, J Weir McCall, C Xie, M Xu

Omics

L V Wain (*Co-Lead)*, J K Baillie (*Co-Lead*), H Baxendale, C E Brightling, M Brown, J D Chalmers, R A Evans, B Gooptu, W Greenhalf, H E Hardwick, R G Jenkins, D Jones, I Koychev, C Langenberg, A Lawrie, P L Molyneaux, A Shikotra, J Pearl, M Ralser, N Sattar, R M Saunders, J T Scott, T Shaw, D Thomas, D Wilkinson

Working Groups

Airways

L G Heaney (*Co-Lead*), A De Soyza (*Co-Lead*), D Adeloye, C E Brightling, J S Brown, J Busby, J D Chalmers, C Echevarria, L Daines, O Elneima, RA Evans, J Hurst, P Novotny, C Nicolaou, P Pfeffer, K Poinasamy, J Quint, I Rudan, E Sapey, M Shankar-Hari, A Sheikh, S Siddiqui, S Walker, B Zheng

Brain

J R Geddes (*Lead*), M Hotopf *(Co-Lead),* K Abel, R Ahmed, L Allan, C Armour, D Baguley, D Baldwin, C Ballard, K Bhui, G Breen, K Breeze, M Broome, T Brugha, E Bullmore, D Burn, F Callard, J Cavanagh, T Chalder, D Clark, A David, B Deakin, H Dobson, B Elliott, J Evans, RA Evans, R Francis, E Guthrie, P Harrison, M Henderson, A Hosseini, N Huneke, M Husain, T Jackson, I Jones, T Kabir, P Kitterick, A Korszun, I Koychev, J Kwan, A Lingford-Hughes, P Mansoori, H McAllister-Williams, K McIvor, B Michael, L Milligan, R Morriss, E Mukaetova-Ladinska, K Munro, A Nevado-Holgado, T Nicholson, C Nicolaou, S Paddick, C Pariante, J Pimm, K Saunders, M Sharpe, G Simons, J P Taylor, R Upthegrove, S Wessely

Cardiac

G P McCann (*Lead*), S Amoils, C Antoniades, A Banerjee, A Bularga, C Berry, P Chowienczyk, J P Greenwood, A D Hughes, K Khunti, C Lawson, N L Mills, A J Moss, S Neubauer, B Raman, A N Sattar, C L Sudlow, M Toshner,

Immunology

P J M Openshaw (*Lead*), D Altmann, J K Baillie, R Batterham, H Baxendale, N Bishop, C E Brightling, P C Calder, C M Efstathiou, R A Evans, J L Heeney, T Hussell, P Klenerman, F Liew, J M Lord, P Moss, S L Rowland-Jones, W Schwaeble, M G Semple, R S Thwaites, L Turtle, L V Wain, S Walmsley, D Wraith

Intensive Care

M J Rowland (*Lead*), A Rostron (*Co-Lead*), J K Baillie, B Connolly, A B Docherty, N I Lone, D F McAuley, D Parekh, A Rostron, J Simpson, C Summers

Lung Fibrosis

R G Jenkins (*Co-Lead*), J Porter (*Co-Lead*), R J Allen, R Aul, J K Baillie, S Barratt, P Beirne, J Blaikley, R C Chambers, N Chaudhuri, C Coleman, E Denneny, L Fabbri, P M George, M Gibbons, F Gleeson, B Gooptu, B Guillen-Guio, I Hall, N A Hanley, L P Ho, E Hufton, J Jacob, I Jarrold, G Jenkins, S Johnson, M G Jones, S Jones, F Khan, P Mehta, J Mitchell, P L Molyneaux, J E Pearl, K Piper Hanley, K Poinasamy, J Quint, D Parekh, P Rivera-Ortega, L C Saunders, M G Semple, J Simpson, D Smith, M Spears, L G Spencer, S Stanel, I Stewart, A A R Thompson, D Thickett, R Thwaites, L V Wain, S Walker, S Walsh, J M Wild, D G Wootton, L Wright

Metabolic

S Heller (*Co-Lead*), M J Davies (*Co-Lead*), H Atkins, S Bain, J Dennis, K Ismail, D Johnston, P Kar, K Khunti, C Langenberg, P McArdle, A McGovern, T Peto, J Petrie, E Robertson, N Sattar, K Shah, J Valabhji, B Young

Pulmonary and Systematic Vasculature

L S Howard (*Co-Lead*), Mark Toshner (*Co-Lead*), C Berry, P Chowienczyk, A Lawrie, O C Leavy, J Mitchell, J Newman, L Price, J Quint, A Reddy, J Rossdale, N Sattar, C Sudlow, A A R Thompson, J M Wild, M Wilkins

Rehabilitation, Sarcopenia and Fatigue

S J Singh (*Co-Lead*), W D-C Man (*Co-Lead*), J M Lord (*Co-Lead*), N J Greening (*Co-Lead*), T Chalder (*Co-Lead*), J T Scott (*Co-Lead*), N Armstrong, E Baldry, M Baldwin, N Basu, M Beadsworth, L Bishop, C E Bolton, A Briggs, M Buch, G Carson, J Cavanagh, H Chinoy, C Dawson, E Daynes, S Defres, R A Evans, L Gardiner, P Greenhaff, S Greenwood, M Harvie, L HOuchen-Wolloff, M Husain, S MacDonald, A McArdle, H J C McAuley, A McMahon, M McNarry, G Mills, C Nolan, K O'Donnell, D Parekh, Pimm, J Sargent, L Sigfrid, M Steiner, D Stensel, A L Tan, I Vogiatzis, J Whitney, D Wilkinson, D Wilson, M Witham, D G Wootton, T Yates

Renal

D Thomas (*Lead*), N Brunskill (*Co-Lead*), S Francis (*Co-Lead*), S Greenwood (*Co-Lead*), C Laing (*Co-Lead*), K Bramham, P Chowdhury, A Frankel, L Lightstone, S McAdoo, K McCafferty, M Ostermann, N Selby, C Sharpe, M Willicombe

Patient Public Engagement Group

L Houchen-Wolloff (*Lead*), J Bunker, R Gill, C Hastie, R Nathu, N Rogers, N Smith

Local Clinical Centre PHOSP-COVID trial staff

(listed in alphabetical order)

Airedale NHS Foundation Trust

A Shaw (PI), L Armstrong, B Hairsine, H Henson, C Kurasz, L Shenton

Aneurin Bevan University Health Board

S Fairbairn (PI), A Dell, N Hawkings, J Haworth, M Hoare, A Lucey, V Lewis, G Mallison, H Nassa, C Pennington, A Price, C Price, A Storrie, G Willis, S Young

Barts Health NHS Trust & Queen Mary University of London

P Pfeffer (PI), K Chong-James, C David, W Y James, C Manisty, A Martineau, O Zongo

Barnsley Hospital NHS Foundation Trust

A Sanderson (PI)

Belfast Health and Social Care Trust & Queen's University Belfast

L G Heaney (PI), C Armour, V Brown, T Craig, S Drain, B King, N Magee, D McAulay, E Major, L McGarvey, J McGinness, R Stone

Betsi Cadwaladr University Health Board

A Haggar (PI), A Bolger, F Davies, J Lewis, A Lloyd, R Manley, E McIvor, D Menzies, K Roberts, W Saxon, D Southern, C Subbe, V Whitehead

Borders General Hospital, NHS Borders

H El-Taweel (PI), J Dawson, L Robinson

Bradford Teaching Hospitals NHS Foundation Trust

D Saralaya (PI), L Brear, K Regan, K Storton

Cambridge University Hospitals NHS Foundation Trust, NIHR Cambridge Clinical Research Facility & University of Cambridge

J Fuld (PI), A Bermperi, I Cruz, K Dempsey, A Elmer, H Jones, S Jose, S Marciniak, M Parkes, C Ribeiro, J Taylor, M Toshner, L Watson, J Weir McCall, J Worsley

Cardiff and Vale University Health Board

R Sabit (PI), L Broad, A Buttress, T Evans, M Haynes, L Jones, L Knibbs, A McQueen, C Oliver, K Paradowski, J Williams

Chesterfield Royal Hospital NHS Trust

E Harris (PI), C Sampson

Cwm Taf Morgannwg University Health Board

C Lynch (PI), E Davies, C Evenden , A Hancock, K Hancock, M Rees , L Roche, N Stroud, T Thomas-Woods

East Cheshire NHS Trust

M Babores (PI), J Bradley-Potts, M Holland, N Keenan, S Shashaa, H Wassall

East Kent Hospitals University NHS Foundation Trust

E Beranova (PI), H Weston (PI), T Cosier, L Austin, J Deery, T Hazelton, C Price, H Ramos, R Solly, S Turney

Gateshead NHS Trust

L Pearce (PI), W McCormick, S Pugmire, W Stoker, A Wilson

Guy's and St Thomas' NHS Foundation Trust

N Hart (PI), LA Aguilar Jimenez, G Arbane, S Betts, K Bisnauthsing, A Dewar, P Chowdhury, A Chiribiri, A Dewar, G Kaltsakas, H Kerslake, MM Magtoto, P Marino, LM Martinez, C O'Brien, M Ostermann, J Rossdale, TS Solano, E Wynn

Hampshire Hospitals NHS Foundation Trust

N Williams (PI), W Storrar (PI), M Alvarez Corral, A Arias, E Bevan, D Griffin, J Martin, J Owen, S Payne, A Prabhu, A Reed, C Wrey Brown

Harrogate and District NHD Foundation Trust

C Lawson (PI), T Burdett, J Featherstone, A Layton, C Mills, L Stephenson,

Hull University Teaching Hospitals NHS Trust & University of Hull

N Easom (PI), P Atkin, K Brindle, M G Crooks, K Drury, R Flockton, L Holdsworth, A Richards, D L Sykes, S Thackray-Nocera, C Wright

Hywel Dda University Health Board

K E Lewis (PI), A Mohamed (PI), G Ross (PI), S Coetzee, K Davies, R Hughes, R Loosley, L O'Brien, Z Omar, H McGuinness, E Perkins, J Phipps, A Taylor, H Tench, R Wolf-Roberts

Imperial College Healthcare NHS Trust & Imperial College London

L S Howard (PI), O Kon (PI), D C Thomas (PI), S Anifowose, L Burden, E Calvelo, B Card, C Carr, E R Chilvers, D Copeland, P Cullinan, P Daly, C M Efstathiou, L Evison, T Fayzan, H Gordon, S Haq, R G Jenkins, C King, F Liew, K March, M Mariveles, L McLeavey, N Mohamed, S Moriera, U Munawar, J Nunag, U Nwanguma, L Orriss- Dib, D P O'Regan, A Ross, M Roy, E Russell, K Samuel, J Schronce, N Simpson, L Tarusan, C Wood, N Yasmin

Kettering General Hospital NHS Trust

R Reddy (PI), A-M, Guerdette, M Hewitt, K Warwick, S White

King's College Hospital NHS Foundation Trust & Kings College London

A M Shah (PI), C J Jolley (PI), O Adeyemi, R Adrego, H Assefa-Kebede, J Breeze, M Brown, S Byrne, T Chalder, A Chiribiri, P Dulawan, N Hart, A Hayday, A Hoare, A Knighton, M Malim, C O'Brien, S Patale, I Peralta, N Powell, A Ramos, K Shevket, F Speranza, A Te

Leeds Teaching Hospitals & University of Leeds

P Beirne (PI), A Ashworth, J Clarke, C Coupland, M Dalton, E Wade, C Favager, J Greenwood, J Glossop, L Hall, T Hardy, A Humphries, J Murira, D Peckham, S Plein, J Rangeley, G Saalmink, A L Tan, B Whittam, N Window, J Woods,

Lewisham & Greenwich NHS Trust

G Coakley (PI)

Liverpool University Hospitals NHS Foundation Trust & University of Liverpool

D G Wootton (PI), L Turtle (PI), L Allerton, AM All, M Beadsworth, A Berridge, J Brown, S Cooper, A Cross, D J Cuthbertson, S Defres, S L Dobson, J Earley, N French, W Greenhalf, H E Hardwick, K Hainey, J Hawkes, V Highett, S Kaprowska, G J Kemp, AL Key, S Koprowska, L Lavelle-Langham, N Lewis-Burke, G Madzamba, F Malein, S Marsh, C Mears, L Melling, M J Noonan, L Poll, J Pratt, E Richardson, A Rowe, M G Semple, V Shaw, K A Tripp, B Vinson, L O Wajero, S A Williams-Howard, J Wyles

London North West University Healthcare NHS Trust

S N Diwanji (PI), P Papineni (PI), S Gurram, S Quaid, G F Tiongson, E Watson

Manchester University NHS Foundation Trust & University of Manchester

B Al-Sheklly (PI), A Horsley (PI), C Avram, P Barran, J Blaikely, M Buch, N Choudhury, D Faluyi, T Felton, T Gorsuch, N A Hanley, T Hussell, Z Kausar, C A Miller, N Odell, R Osbourne, K Piper Hanley, K Radhakrishnan, S Stockdale, D Trivedi

Newcastle upon Tyne Hospitals NHS Foundation Trust & University of Newcastle

A De Soyza (PI), C Echevarria (PI), A Ayoub, J Brown, G Burns, G Davies, H Fisher, C Francis, A Greenhalgh, P Hogarth, J Hughes, K Jiwa, G Jones, G MacGowan, D Price, A Sayer, J Simpson, H Tedd, S Thomas, S West, M Witham, S Wright, A Young

NHS Dumfries and Galloway

M J McMahon (PI), P Neill

NHS Greater Glasgow and Clyde Health Board & University of Glasgow

D Anderson (PI), H Bayes (PI), C Berry (PI), D Grieve (PI), I B McInnes (PI), N Basu, A Brown, A Dougherty, K Fallon, L Gilmour, K Mangion, A Morrow, K Scott, R Sykes, R Touyz

NHS Highland

E K Sage (PI), F Barrett, A Donaldson

NHS Lanarkshire

M Patel (PI), D Bell, A Brown, M Brown, R Hamil, K Leitch, L Macliver, J Quigley, A Smith, B Welsh

NHS Lothian & University of Edinburgh

G Choudhury (PI), J K Baillie, S Clohisey, A Deans, A B Docherty, J Furniss, E M Harrison, S Kelly, N I Lone, D E Newby, A Sheikh

NHS Tayside & University of Dundee

J D Chalmers (PI), D Connell, A Elliott, C Deas, J George, S Mohammed, J Rowland, A R Solstice, D Sutherland, C J Tee

North Bristol NHS Trust & University of Bristol

N Maskell (PI), D Arnold, S Barrett, H Adamali, A Dipper, S Dunn, A Morley, L Morrison, L Stadon, S Waterson, H Welch

North Middlesex Hospital NHS Trust

B Jayaraman (PI), T Light

Nottingham University Hospitals NHS Trust & University of Nottingham

C E Bolton (PI), P Almeida, J Bonnington, M Chrystal, E Cox, C Dupont, S Francis, P Greenhaff, A Gupta, L Howard, W Jang, S Linford, L Matthews, R Needham, A Nikolaidis, S Prosper, K Shaw, A K Thomas

Oxford University Hospitals NHS Foundation Trust & University of Oxford

L P Ho (PI), N M Rahman (PI), M Ainsworth, A Alamoudi, M Beggs, A Bates, A Bloss, A Burns, P Carter, M Cassar, K M Channon, J Chen, F Conneh, T Dong, R I Evans, E Fraser, X Fu, J R Geddes, F Gleeson, P Harrison, M Havinden-Williams, P Jezzard, N Kanellakis, I Koychev, P Kurupati, X Li, E Lukaschuk, K McGlynn, H McShane, C Megson, K Motohashi, S Neubauer, D Nicoll, G Ogg, E Pacpaco, M Pavlides, Y Peng, N Petousi, J Propescu, N Rahman, B Raman, M J Rowland, K Saunders, M Sharpe, N Talbot, E Tunnicliffe

Royal Brompton and Harefield Clinical Group, Guy’s and St Thomas’ NHS Foundation Trust.

W D-C Man (PI), B Patel (PI), R E Barker, D Cristiano, N Dormand, M Gummadi, S Kon, K Liyanage, C M Nolan, S Patel, O Polgar, P Shah, S J Singh, J A Walsh

Royal Free London NHS Foundation Trust

J Hurst (PI), H Jarvis (PI), S Mandal (PI), S Ahmad, S Brill, L Lim, D Matila, O Olaosebikan, C Singh

Royal Papworth Hospital NHS Foundation Trust

M Toshner (PI), H Baxendale, L Garner, C Johnson, J Mackie, A Michael, J Pack, K Paques, H Parfrey, J Parmar

Salford Royal NHS Foundation Trust

N Diar Bakerly (PI), P Dark, D Evans, E Hardy, A Harvey, D Holgate, S Knight, N Mairs, N Majeed, L McMorrow, J Oxton, J Pendlebury, C Summersgill, R Ugwuoke, S Whittaker

Salisbury NHS Foundation Trust

W Matimba-Mupaya (PI), S Strong-Sheldrake

Sheffield Teaching NHS Foundation Trust & University of Sheffield

S L Rowland-Jones (PI), A A R Thompson (Co PI), J Bagshaw, M Begum, K Birchall, R Butcher, H Carborn, F Chan, K Chapman, Y Cheng, L Chetham, C Clark, Z Coburn, J Cole, M Dixon, A Fairman, J Finnigan, L Finnigan, H Foot, D Foote, A Ford, R Gregory, K Harrington, L Haslam, L Hesselden, J Hockridge, A Holbourn, B Holroyd-Hind, L Holt, A Howell, E Hurditch, F Ilyas, C Jarman, A Lawrie, E Lee, J-H Lee, R Lenagh, A Lye, I Macharia, M Marshall, A Mbuyisa, J McNeill, S Megson, J Meiring, L Milner, S Misra, H Newell, T Newman, C Norman, L Nwafor, D Pattenadk, M Plowright, J Porter, P Ravencroft, C Roddis, J Rodger, P Saunders, J Sidebottom, J Smith, L Smith, N Steele, G Stephens, R Stimpson, B Thamu, N Tinker, K Turner, H Turton, P Wade, S Walker, J Watson, J M Wild, I Wilson, A Zawia

St George's University Hospitals NHS Foundation Trust

R Aul (PI), M Ali, A Dunleavy (PI), D Forton, N Msimanga, M Mencias, T Samakomva, S Siddique, J Teixeira, V Tavoukjian

Sherwood Forest Hospitals NHS Foundation Trust

J Hutchinson (PI), L Allsop, K Bennett, P Buckley, M Flynn, M Gill, C Goodwin, M Greatorex, H Gregory, C Heeley, L Holloway, M Holmes, J Kirk, W Lovegrove, TA Sewell, S Shelton, D Sissons, K Slack, S Smith, D Sowter, S Turner, V Whitworth, I Wynter

Shropshire Community Health NHS Trust

L Warburton (PI), S Painter, J Tomlinson

Somerset NHS Foundation Trust

C Vickers (PI), T Wainwright, D Redwood, J Tilley, S Palmer

Swansea Bay University Health Board

G A Davies (PI), L Connor, A Cook, T Rees, F Thaivalappil, C Thomas

Tameside and Glossop Integrated Care NHS Foundation

A Butt (PI), M Coulding, H Jones, S Kilroy, J McCormick, J McIntosh, H Savill, V Turner, J Vere

The Great Western Hospital Foundation Trust

E Fraile (PI), J Ugoji

The Hillingdon Hospitals NHS Foundation Trust

S S Kon (PI), H Lota, G Landers, M Nasseri, S Portukhay

The Rotherham NHS Foundation Trust

A Hormis (PI), A Daniels, J Ingham, L Zeidan

United Lincolnshire Hospitals NHS Trust

M Chablani (PI), L Osborne

University College London Hospital & University College London

M Marks (PI), J S Brown (PI), N Ahwireng, B Bang, D Basire, R C Chambers, A Checkley, R Evans, M Heightman, T Hillman, J Hurst, J Jacob, S Janes, R Jastrub, M Lipman, S Logan, D Lomas, M Merida Morillas, A Pakzad, H Plant, J C Porter, K Roy, E Wall, B Williams, M Xu

University Hospital Birmingham NHS Foundation Trust & University of Birmingham

D Parekh (PI), N Ahmad Haider, C Atkin, R Baggott, M Bates, A Botkai, A Casey, B Cooper, J Dasgin, K Draxlbauer, N Gautam, J Hazeldine, T Hiwot, S Holden, K Isaacs, T Jackson, S Johnson, V Kamwa, D Lewis, J M Lord, S Madathil, C McGhee, K Mcgee, A Neal, A Newton Cox, J Nyaboko, D Parekh, Z Peterkin, H Qureshi, B Rangelov, L Ratcliffe, E Sapey, J Short, T Soulsby, R Steeds, J Stockley, Z Suleiman, T Thompson, M Ventura, S Walder, C Welch, D Wilson, S Yasmin, K P Yip

University Hospitals of Derby and Burton

P Beckett (PI) C Dickens, U Nanda

University Hospitals of Leicester NHS Trust & University of Leicester

C E Brightling (CI), R A Evans (PI), M Aljaroof, N Armstrong, H Arnold, H Aung, M Bakali, M Bakau, M Baldwin, M Bingham, M Bourne, C Bourne, N Brunskill, P Cairns, L Carr, A Charalambou, C Christie, M J Davies, S Diver, S Edwards, C Edwardson, O Elneima, H Evans, J Finch, S Glover, N Goodman, B Gootpu, N J Greening, B Guillen-Guio, K Hadley, P Haldar, B Hargadon, V C Harris, L Houchen-Wolloff, W Ibrahim, L Ingram, K Khunti, A Lea, D Lee, D Lozano-Rojas, G P McCann, H J C McAuley, P McCourt, T Mcnally, G Mills, A Moss, W Monteiro, K Ntotsis, M Pareek, S Parker, A Rowland, A Prickett, I N Qureshi, R Russell, N Samani, M Sereno, M Sharma, A Shikotra, S Siddiqui, A Singapuri, S J Singh, J Skeemer, M Soares, E Stringer, T Thornton, M Tobin, E Turner, L V Wain, T J C Ward, F Woodhead, J Wormleighton, T Yates, A Yousuf,

University Hospital Southampton NHS Foundation Trust & University of Southampton

M G Jones (PI), C Childs, R Djukanovic, S Fletcher, M Harvey, E Marouzet, B Marshall, R Samuel, T Sass, T Wallis, H Wheeler

Whittington Health NHS
R Dharmagunawardena (PI), E Bright, P Crisp, M Stern

Wirral University Teaching Hospital
A Wight (PI), L Bailey, A Reddington

Wrightington Wigan and Leigh NHS trust
A Ashish (PI), J Cooper, E Robinson

Yeovil District Hospital NHS Foundation Trust
A Broadley (PI)

York & Scarborough NHS Foundation Trust
K Howard (PI), L Barman, C Brookes, K Elliott. L Griffiths, Z Guy, D Ionita, H Redfearn, C Sarginson A Turnbull

Health and Care Research Wales
Y Ellis

London School of Hygiene & Tropical Medicine (LSHTM)
M Marks, A Briggs

NIHR Office for Clinical Research Infrastructure
K Holmes

Patient Public Involvement Leads
Asthma UK and British Lung Foundation Partnership - K Poinasamy, S Walker

Royal Surrey NHS Foundation Trust
M Halling-Brown

South London and Maudsley NHS Foundation Trust & Kings College London
G Breen, M Hotopf

Swansea University & Swansea Welsh Network
K Lewis, N Williams

Supplementary methods

PHOSP-COVID study and timeline

We recruited participants from the Post-HOSPitalisation COVID-19 study (PHOSP-COVID), which is a large-scale long-term study of nearly 8,000 adults discharged from one of 83 participating UK National Health Service (NHS) hospitals with a clinical diagnosis of COVID-19 (between February 1, 2020 and March 31, 2021).[1,2] A subset of participants (n=2,697) underwent additional specific research visits alongside routine clinical care and provided data at baseline (i.e. during hospitalisation), at 6-months post-admission (2-7 months post-discharge) and 12 months after admission. These are referred to as Tier 2 participants. Collected measurements included routine clinical data on admission, results of blood tests on admission, and clinical scales. Tier 2 participants who provided a MoCA at 6-months are those included in our previous study identifying biocognive profiles.[3]

C-Fog is a Tier 3 PHOSP-COVID study, for which a total of 2,469 participants consented to be recontacted for other research and were invited to complete an online computerised cognitive test, clinical scales and an assessment of their occupation. These 2,460 participants only partially overlap with the 2,697 mentioned above (Figure S1). Invitations were sent between November 30, 2022 and December, 7 2022 with a single reminder sent between February 24 and February 28, 2023. Participants completed the tests and questionnaires once, up until May 1, 2023 which corresponded to a time since COVID-19 hospital admission ranging from 21 to 38 months which we refer to as the 2-3 years follow-up. Unlike the other two follow-ups (at 6 and 12 months), the date of invitation for this third follow-up was fixed for all participants and was not based on their time of admission. This is why we refer to it as "2-3 years" to represent the wider range of follow-up time achieved.

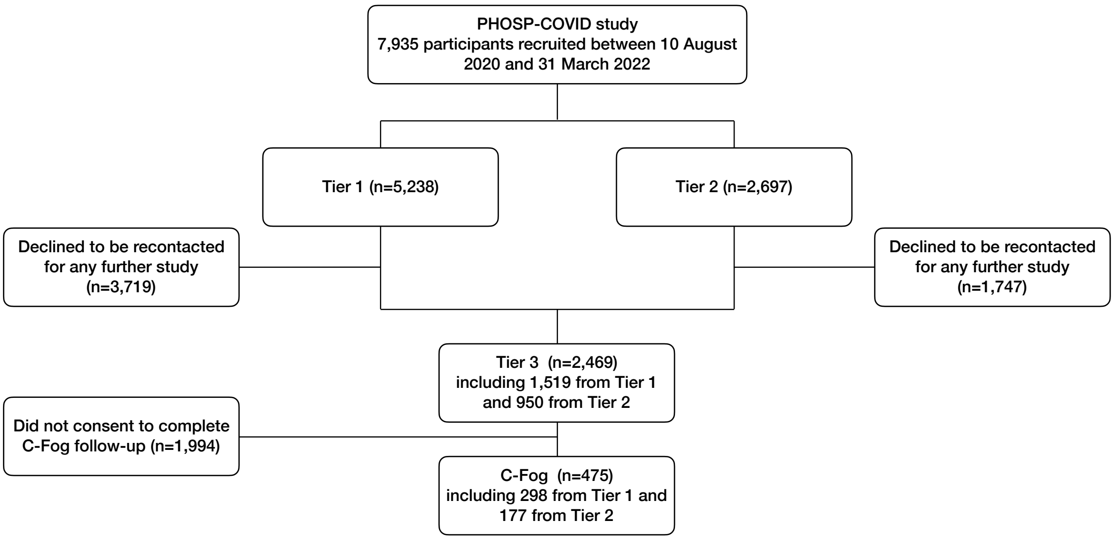

Figure S1 – STROBE diagram of this study (C-Fog) in relation to its parent study (PHOSP-COVID) and its 3 Tiers.

Variables measured at 6 and 12 months

Participants provided a range of data at the 6 and 12 months follow-up which are described elsewhere.[1,2,4] Here we used the following variables:

1) **PHQ-9**: A clinical scale measuring depression

2) **GAD-7**: A clinical scale measuring anxiety

3) **FACIT-Fatigue** (which we refer to as FACIT for short): A clinical scale measuring fatigue

4) **Cognitive subset of the Patient Symptom Questionnaire (C-PSQ)**: a score going from 0 to 7 which assesses a participant's subjective cognitive deficits based on self-reported impairment in seven domains: confusion, short term memory loss, difficulty communicating, difficulty understanding or being understood, difficulty concentrating, slowing down of thinking and difficulty remembering.[3,5]

5) **Montreal Cognitive Assessment** (**MoCA**): A pen and paper cognitive test

6) **Recovery clusters**: these were defined in a previous study[1] using validated objective measures of breathlessness, fatigue, anxiety, depression, post-traumatic stress disorder, physical performance and cognitive impairments at the 6-months follow-up. Four clusters were identified in this study, labelled 'very severe', 'severe', 'moderate with cognitive impairment', and 'mild'. In this study, the 2nd and 3rd clusters were merged into one for two reasons. First, the 'moderate with cognitive impairment' cluster was underrepresented in those followed-up at 2-3 years (with fewer than 25 respondents), which might have led to imprecise estimates of associations. Second, ordering the 2nd and 3rd clusters would be arbitrary, while merging them creates three ordered levels enabling to treat the cluster variable as an ordinal variable in regressions, thus simplifying the model, reducing the number of degrees of freedom, and increasing statistical power.

7) **Biocognitive profiles**: these were defined in a previous study of the PHOSP-COVID cohort.[3] They link profiles of acute blood biomarkers (measured during admission to hospital) and cognitive deficits at the 6-month follow-up. Two biocognitive profiles were identified in that study: a first linking acutely raised fibrinogen with relatively low CRP (i.e. lower compared to that expected for the fibrinogen level) to both objective and subjective cognitive deficits at 6 months; a second linking acutely raised D-dimer relative to CRP to subjective cognitive

deficits. These profiles were identified using canonical correlation analysis. In this study, we used the average canonical variables (referred to as the mean profile in our previous study[3]) to represent each biocognitive profile.

Variables measured at 2-3 years

Variables measured at 2-3 years have not been described elsewhere as they were collected specifically as part of C-Fog. Here we describe them in detail.

After completing an online consent form, participants were invited to complete a set of cognitive tasks in the following order. Each task started with a practice run (whose score is not recorded) to familiarise the participant with the task, followed by the actual recorded task.

1) **Object memory (immediate)**: 20 images are displayed with an encoding time of 2000ms and inter stimulus interval of 500ms. After the full sequence is finished, each stimulus is then presented in turn within a grid of 7 distractors. The distractors are categorised by how similar they are to the stimulus along 3 dimensions.

   The dimensions are:
   - Item: Whether the image is of the same item as the target.
   - Precision: Whether the image is of the same pose or artistic style as the target.
   - Mis-binding: Whether the image is flipped left or right.

   The main outcome measure is the number of points accrued, where points are awarded for each trial as incorrect item = 0, correct item = 1, correct item + precision = 2, correct item, precision and binding = 3, summed across trials.

2) **Simple reaction speed**: The participant is presented with a string of targets, they must click anywhere on the screen as soon as the target appears. The primary score is the median response time.

3) **2D Manipulation**: A target grid of coloured blocks is displayed above a set of 4 similar grids of coloured blocks. Three of the grids have been subtly altered from the target and the 4th is identical to the target. The grids have all been randomly oriented at 90, 180 or 270 degrees from the target grid. The volunteer must click the grid that is identical to the target as quickly and accurately as possible. They have 3 minutes to complete as many trials as they can. The main outcome measure is the total number of correctly identified matches divided by the total

number of trials completed.

4) **Cognitive control** (Switching Stroop): The participant is presented with a coloured block (either red or blue) and on either side the words “red” and “blue”, these words are drawn with either red or blue ink. At the top of the task is a condition, the condition reads either “ink” or “text”. If the condition reads “ink” then the participant must click on the word that has the same ink colour as the square in the middle. If the condition reads “text” then the participant must click on the word that describes the colour of the square in the middle. The condition switches pseudo-randomly as the task progresses. The primary score is the overall response accuracy.

5) **Spatial working memory**: A grid of 16 squares is presented. A sequence of these squares lights up, with each square lit up for 1500ms with an inter-stimulus interval of 0ms. The volunteer must remember the sequence and when it is finished, must click the sequence of squares in the correct order. If the volunteer gets a trial correct, then the number of squares in the sequence increases by 1. If the volunteer fails on a sequence of the same number of items 3 times in a row, the game ends. The main outcome measure is the maximum length sequence correctly remembered by the volunteer.

6) **Spatial planning** (Tower of London): The volunteer is presented with 10 sets of 2 rows of pegs with coloured rings on them. They must identify the minimum number of moves that it would take to transform the top row of pegs to the same configuration as the bottom row of pegs. They must do this in their head and cannot use trial and error to move the rings around. The main outcome measure is the total number of correct trials divided by the total number of trials.

7) **Verbal analogies**: The volunteer is presented with a set of comparative analogies, for example “light is to heavy as fast is to slow”. The volunteer must work out if the comparison is correct. In the given example, this comparison is correct as they are both opposites of each other. An example of an incorrect analogical comparison would be “human is to ape as fast is to slow”. The volunteer must complete as many comparisons as possible in 3 minutes as accurately as possible. The main outcome measure is the total number of correct trials divided by the total number of completed trials.

8) **Object memory (delayed)**: The volunteer is presented with the same task as the Object Memory (Immediate Memory) task described above. However the encoding period is removed. This means the volunteer must remember the stimuli from the beginning of the

battery. The main outcome measure is the total number of points accrued as described in the Object Memory (Immediate Memory).

After the cognitive tasks, clinical scales/questionnaires appeared on the screen which they were invited to complete. These include:

1) PHQ-9
2) GAD-7
3) Occupation questionnaire (see next section)
4) FACIT scale
5) CCI-20 scale for subjective cognitive decline,[15] but modified so that the questions referred to participants' perceived decline compared to before they had COVID-19 rather than compared to five years ago.

Quality control of responses to cognitive tasks

A set of predefined quality checks was applied to responses to the cognitive tasks and individual responses were removed if they did not meet the quality standards. This was done on a per-task basis for each individual. Specifically, individual responses were removed if any of the following applied:

1. The participant had an abnormally fast reaction time, with a task-specific cut-off determined by fitting bimodal distributions to the response time for that task in the normative database, and using the minimum of the bimodal density function between the two modes as the cut-off point.
2. The participant responded very repetitively, i.e. they pressed the same button for over 50% of the trials in a row
3. The participant did not respond at all and the task timed out.
4. The participant left the testing screen during the time they were completing the task.
5. The participant took a very long time (over 5 standard deviations longer than the mean in the normative population) to finish a task.
6. There was evidence of machine error, i.e. scores that were theoretically impossible (e.g. higher than the maximum possible score) or 10 standard deviations away from the mean of the data.

Normative model for cognitive tasks

We used normative data from the Great British Intelligence test study,[7] an ongoing study of cognitive function in the general population in the UK with self-selected volunteers. Volunteers with self-

reported neurological or psychiatric conditions were excluded to build the normative model. Within this study, different tasks were added to the data collection at different times and so the number of participants who provided normative data varied between tasks, as follows:

Object memory (immediate): 43,250 participants

Simple reaction speed: 5,796 participants

2D manipulation: 332,553 participants

Cognitive control: 19,199 participants

Spatial working memory: 349,587 participants

Spatial planning: 19,328 participants

Verbal analogies: 20,734 participants

Object memory (delayed) = 43,380 participants

Using data from the normative population, for each task, a linear regression model was estimated in which the score of the task was the dependent variable, and age, age^2, sex, ethnicity, educational level, and whether language was the participant's main language were the independent variables. The coefficients of that linear regression were then used to adjust the scores for participants in our study. The adjusted scores were then divided by the standard deviation of the normative population to obtain z-scores.

Predefined thresholds on clinical scales and cognitive tests

Thresholds were applied on the scores of clinical scales and cognitive tests to define 'No symptom', 'Mild symptoms, 'Moderate symptoms', and 'Severe symptoms'. These are colour-coded in Figure 1. For PHQ-9 and GAD-7, we used standard thresholds (≥ 5 for mild, ≥ 10 for moderate, ≥ 15 for severe).

For FACIT, we used published general population norms (population mean 43.5, SD: 8.3)[8] and defined mild symptom as a score at least 1 SD below the mean (i.e. 43.5-8.3), moderate impairment as 2 SD below the mean and severe impairment as 3 SD below the mean.

For CCI-20, we built on thresholds commonly recommended for the first 12 items and scaled them up to 20 items. Specifically, a cut-off of 15 or less on the first 12 items used to be considered as an indication of no cognitive decline[9] and a cut-off of 20 or more is now preferred to define the presence of decline.[10] We interpret values between these two cut-offs (which, scaled up to the 20 items, equate to 25 to 35) to represent mild impairment. By extension, we then define 35-45 to represent moderate decline, and a score above 45 to represent severe decline.

For thresholds on the z-scores of cognitive tasks and overall cognitive score, we used 1 SD below the mean to define mild impairment, 1.5 SD to define moderate impairment, and 2 SD to define severe impairment.

Assessment of occupation change

Occupation change was determined based on the following questions:

1) Please describe your occupation/working status today
    a. Working full-time
    b. Working part-time
    c. Full-time carer (children or other)
    d. Unemployed
    e. Unable to work due to chronic illness
    f. Student
    g. Retired
    h. Medically retired
    i. Prefer not to say

2) Compared to before your COVID-19 illness, is your main occupation/working status:
    a. Same as before
    b. Different from before
    c. Prefer not to say

3) If different from before, why did your occupation/working status change?
    a. Poor health
    b. New caring responsibility

c. Working hours reduced by employer
d. Made redundant
e. Sick leave
f. Other
g. Prefer not to say

In our analysis (as in a previous study[3]), occupation was deemed to have changed if the participant reported that their occupation had changed between before and after their COVID-19 illness (question 2), if their current occupation (question 1) was not 'working full-time' (as a change in occupation can also reflect an increase in number of hours worked), and for whom there was information on their occupation before they had COVID-19 (based on answers collected in previous study visit).

We separately reported the current occupation (question 1) for those who did and those who did not report occupation change. For this analysis, we merged the answer "Medically retired" and "Unable to work due to chronic illness" because in one subgroup, "medically retired" had too few positive responses and we have an obligation (for privacy reason) to censor results with fewer than 5 responses. Merging the two allowed us to present the whole dataset.

Details about statistical analysis

Lasso regression was computed using glmnet version 4.1.8 in R by setting the alpha parameter to 1. The regularisation parameter (lambda) was optimised using cross-validation (using cv.glmnet) over a log-grid: 10^{-5}, $10^{-4.9}$, $10^{-4.8}$, …, 10^{-1}.

To assess the risk of worsening of existing symptoms, we calculated paired t-tests among individuals with at least mild symptoms at the first time point (i.e. at 6 or 12 months depending on the comparison) and at the second time point (i.e. 2-3 years). To assess the risk of emerging symptoms in a way that is not biased by regression to the mean (which could occur, for instance, by restriction the cohort to those with no symptoms at the first time point), we computed paired t-tests between participants with no symptom at the first time point *and/or* no symptom at the second time point. This analysis treats the two time points symmetrically and is therefore not subject to regression to the mean. It includes people who were well and became symptomatic (emergent cases), people who were symptomatic and became well (remitted cases), and people who were well throughout. The

interpretation of this analysis is therefore intuitive: an average increase in symptom burden indicates a net emergence of symptoms whereas an average decrease indicates a net remission of symptoms.

When predicting symptoms using a range of factors, linear regressions were used. For continuous and binary factors, a linear regression was used. For ordinal factors, the linear trend was assessed and reported. Age, sex, and time since infection were included as covariates. For the prediction based on symptoms, the same symptom domain as the outcome but measured at 6 months was also added (e.g. adjusting for PHQ-9 at 6 months when predicting PHQ-9 at 2-3 years based on GAD-7 at 6 months). Homoscedasticity was tested using the Breusch-Pagan test (see Table S10 below). The linear assumption for continuous independent variables was assessed by visual inspection of the plot of residuals as a function of predicted values.

Wilson's score intervals were used to calculate 95% CI of absolute risks. These provide better coverage than the so-called exact 95% CI.[11]

Supplementary figures

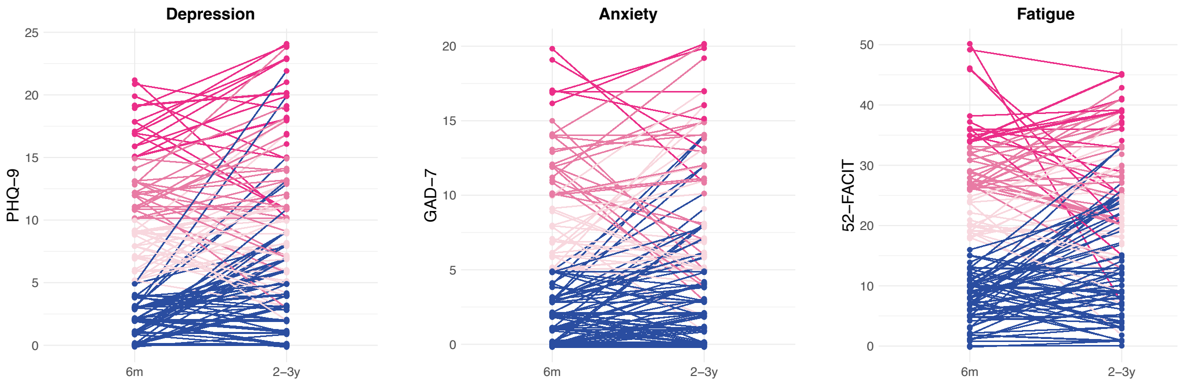

Figure S2 – Paired values of different scales at 6 months and 2-3 years colour-coded by the severity of symptoms at 6 months. Blue, light pink, dark pink and red dots indicate those with no symptom, mild symptoms, moderate symptoms, and severe symptoms respectively. The lines are colour-coded by the severity of the symptoms at 6 months.

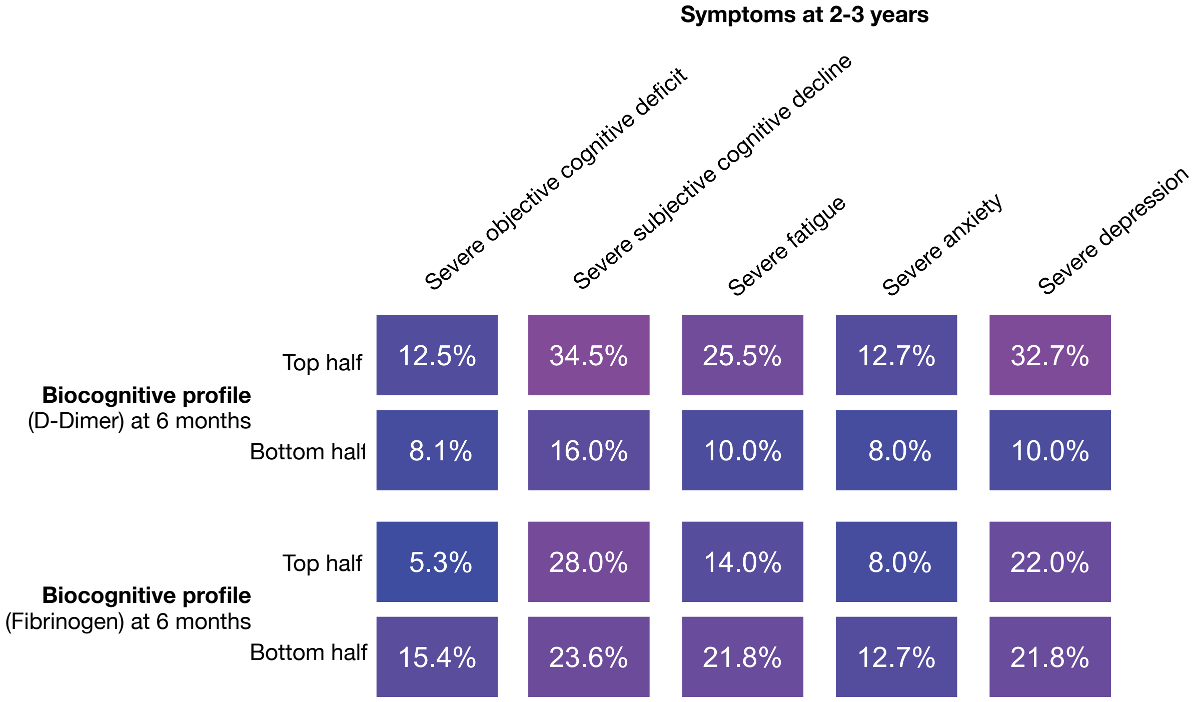

Figure S3 – Prevalence of severe psychiatric, cognitive and fatigue outcomes at 2-3 years as a function of biocognitive profiles at 6 months. Biocognitive profiles are defined as continuous scores linking acute biomarkers with cognitive outcome at 6 months. Here we report the distribution of symptoms at 2-3 years among those in the top half versus bottom half of the cohort in terms of these scores.

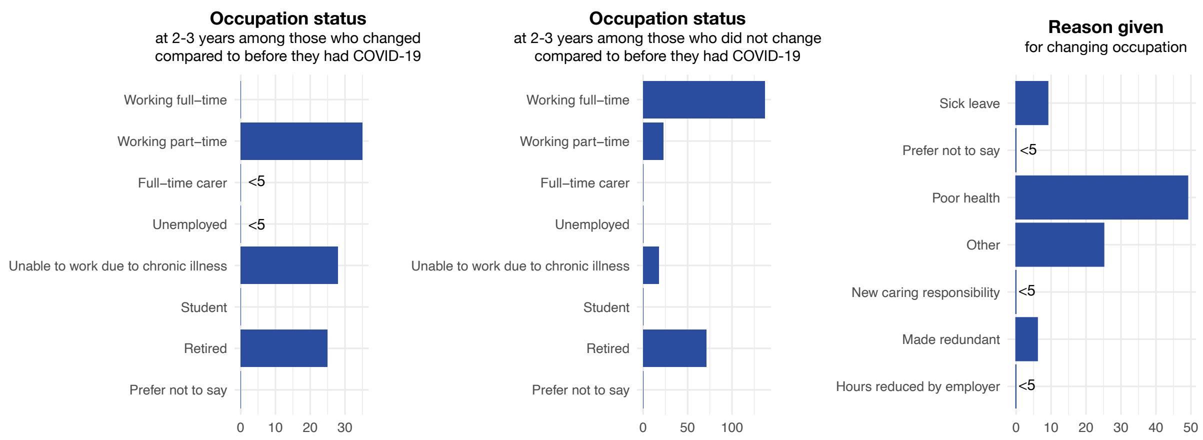

Figure S4 – Occupation status among those who changed and those who did not change occupation compared to before they had COVID-19 and reason given for changing occupation among those who did. For privacy reason, entries with fewer than 5 participants are reported as “<5”.

Supplementary tables

Table S1 – Number of responses for each variable and percentage of the total number of respondents

Item	Number of responses (%)
**Cognitive domain (after quality control)**	
2D Manipulation	431 (90.7)
Object Memory (immediate)	427 (89.9)
Spatial Span	410 (86.3)
Object Memory (delayed)	304 (64)
Simple reaction speed	454 (95.6)
Cognitive control	437 (92)
Spatial Planning	392 (82.5)
Verbal Analogies	403 (84.8)
**Clinical scales at 2-3 years**	
PHQ-9 at 2-3 years	353 (74.3)
GAD-7 at 2-3 years	353 (74.3)
FACIT at 2-3 years	353 (74.3)
CCI-20 at 2-3 years	353 (74.3)
**Clinical scales at 6-months among those with data at 2-3 years**	
PHQ-9 at 6 months among those with data at 2-3 years	172 (36.2)
GAD-7 at 6 months among those with data at 2-3 years	172 (36.2)
FACIT at 6 months among those with data at 2-3 years	150 (31.6)
C-PSQ at 6 months among those with data at 2-3 years	148 (31.2)
MoCA at 6 months among those with data at 2-3 years	151 (31.8)
**Clinical scales at 12 months among those with data at 2-3 years**	
PHQ-9 at 12 months among those with data at 2-3 years	168 (35.4)
GAD-7 at 12 months among those with data at 2-3 years	168 (35.4)
FACIT at 12 months among those with data at 2-3 years	70 (14.7)
C-PSQ at 12 months among those with data at 2-3 years	140 (29.5)
MoCA at 12 months among those with data at 2-3 years	148 (31.2)
**Clinical scales at 6/12 months among those with the same scale at 2-3 years**	
PHQ-9 at 6 months among those PHQ-9 at 2-3 years	130 (27.4)
GAD-7 at 6 months among those GAD-7 at 2-3 years	130 (27.4)
FACIT at 6 months among those FACIT at 2-3 years	117 (24.6)
PHQ-9 at 12 months among those PHQ-9 at 2-3 years	131 (27.6)
GAD-7 at 12 months among those GAD-7 at 2-3 years	131 (27.6)
FACIT at 12 months among those FACIT at 2-3 years	50 (10.5)

Table S2 – Differences in outcomes at 2-3 years between those who responded after the first invitation and those who responded after receiving a reminder.

	**First invitation** Mean score	**Reminder** Mean score	**Difference in mean** 95% CI	**p-value**
**Depression (PHQ-9)**	9.58	10.13	-1.09 – 2.20	0.51
**Anxiety (GAD-7)**	6.23	7.11	-0.48 – 2.24	0.20
**Fatigue (52-FACIT)**	22.16	21.81	-3.71 – 3.00	0.83
**Subjective cognitive deficit (CCI-20)**	30.15	27.40	-7.68 – 2.18	0.27
**Objective cognitive deficit (normalised score in SD)**	-0.64	-0.88	-0.46 – -0.0035	0.047

Table S3 – Results of paired tests for participants who provided data at different time points. Each cell contains the mean change in the symptom domain. A positive value indicates an increase in symptom burden with time (for fatigue, the scale is reversed, i.e. 52-FACIT is reported, so that positive values also indicate worsening of symptoms with time). 95% confidence intervals are in brackets. Tests limited to persistent symptoms refer to participants who had at least mild symptoms burden at both time points. Tests limited to emergent/remitting symptoms refer to participants who had no symptoms at at least one time point.

	**Depression (PHQ-9)**	**Anxiety (GAD-7)**	**Fatigue (52-FACIT)**
**6m to 12m**	-0.72 (-1.19,-0.26), p=0.0023	-0.24 (-0.68, 0.19), p=0.27	-1.69 (-3.10,-0.29), p=0.018
**12m to 2-3 years**	2.12 (1.19,3.05), p<0.0001	0.71 (-0.11, 1.53), p=0.091	3.90 (1.97,5.84), p=0.00012
**12m to 2-3 years (persistent)**	1.78 (0.32,3.24), p=0.018	0.68 (-0.82, 2.18), p=0.37	3.98 (1.61,6.35), p=0.0015
**12m to 2-3 years (emergent/remitting)**	2.40 (1.18,3.63), p=0.00021	0.73 (-0.27, 1.73), p=0.15	3.85 (0.99,6.72), p=0.0092
**6m to 2-3 years**	1.77 (0.95,2.59), p<0.0001	0.82 (0.15,1.48), p=0.017	1.73 (-0.097, 3.549), p=0.063
**6m to 2-3 years (persistent)**	1.74 (0.50,2.99), p=0.0068	0.81 (-0.50, 2.12), p=0.22	1.20 (-1.46, 3.86), p=0.37
**6m to 2-3 years (emergent/remitting)**	1.79 (0.68,2.91), p=0.0021	0.82 (0.058,1.580), p=0.035	2.08 (-0.44, 4.60), p=0.10

Table S4– Incidence and remission proportions for the different outcomes at 2-3 years for symptoms not present (for incidence)/present (for remission) at 6 months. All proportions are in percent. Number in brackets are 95% CI.

Outcome	Incidence (any severity)	Incidence (moderate to severe)	Remission proportion
Depression (PHQ-9)	42.4 (30.6–55.1)	6.8 (2.3–16.8)	6.5 (2.2–16.0)
Anxiety (GAD-7)	19.1 (11.4–30.2)	5.9 (1.9–14.7)	17.3 (9.2–30.0)
Fatigue (FACIT)	25.9 (16.3–38.5)	13.4 (7.5–22.7)	9.8 (3.9–21.5)

Table S5 – Prediction of outcomes by factors representing different earlier aspects of the participant's illness. Each cell in the table contains the coefficient in the regression wherein the outcome is the dependent variable and the predictor (as well as age, sex, and time since infection) are the independent variables. The p-values are reported as uncorrected (p) and Benjamini & Hochberg-corrected (q) for each predictor independently. For ordinal variable (WHO and Recovery cluster), the coefficient represents the change in the dependent variable associated with an increase by one level in the predictor. For instance, PHQ-9 increases by 9.11 on average when going from mild to moderate-to-severe recovery. WHO=World Health Organization Clinical Progression Scale, NEWS = National Early Warning Score, PE=Pulmonary embolism, ICU=Intensive Care Unit, ME=Myalgic Encephalomyelitis, CFS=Chronic fatigue syndrome. There was little evidence of heteroscedasticity (Table S10 below) and non-linearity (data not shown).

	**Depression (PHQ-9)**	**Anxiety (GAD-7)**	**Fatigue (52-FACIT)**	**Subjective cognitive deficit (CCI-20)**	**Objective cognitive deficit (normalised score in SD)**
**WHO**	-1.27 (-2.92 – 0.39), p=0.13, q=0.24	-1.71 (-3.11 – -0.31), p=0.017, q=0.085	-1.49 (-4.73 – 1.76), p=0.37, q=0.37	-3.80 (-8.92 – 1.32), p=0.15, q=0.24	0.15 (-0.086 – 0.39), p=0.21, q=0.27
**NEWS**	0.061 (-0.27 – 0.39), p=0.72, q=0.94	0.032 (-0.24 – 0.31), p=0.82, q=0.94	-0.072 (-0.71 – 0.57), p=0.83, q=0.94	0.039 (-0.96 – 1.04), p=0.94, q=0.94	0.015 (-0.031 – 0.061), p=0.52, q=0.94
**Duration of admission**	-0.004 (-0.039 – 0.031), p=0.82, q=0.91	-0.02 (-0.05 – 0.0092), p=0.18, q=0.76	0.036 (-0.032 – 0.10), p=0.30, q=0.76	0.0061 (-0.10 – 0.11), p=0.91, q=0.91	-0.0012 (-0.0059 – 0.0035), p=0.61, q=0.91
**ICU admission**	-0.87 (-3.22 – 1.49), p=0.47, q=0.62	-1.07 (-2.98 – 0.84), p=0.27, q=0.62	-1.55 (-6.03 – 2.93), p=0.50, q=0.62	-5.20 (-12.57 – 2.18), p=0.17, q=0.62	0.056 (-0.25 – 0.37), p=0.72, q=0.72
**PE**	-0.88 (-3.51 – 1.76), p=0.52, q=0.64	-1.55 (-3.74 – 0.64), p=0.17, q=0.42	0.25 (-4.99 – 5.50), p=0.92, q=0.92	-4.78 (-13.07 – 3.51), p=0.26, q=0.43	0.43 (0.038 – 0.82), p=0.033, q=0.17
**Delirium**	1.05 (-1.80 – 3.90), p=0.47, q=0.97	1.19 (-1.20 – 3.58), p=0.33, q=0.97	0.096 (-5.81 – 6.01), p=0.97, q=0.97	1.05 (-8.02 – 10.13), p=0.82, q=0.97	-0.11 (-0.56 – 0.33), p=0.61, q=0.97
**History of psychiatric/neurological comorbidity**	5.05 (3.53 - 6.58), p<0.0001, q<0.0001	3.30 (1.97 - 4.63), p<0.0001, q<0.0001	8.69 (5.63 - 11.74), p<0.0001, q<0.0001	12.73 (7.90 - 17.55), p<0.0001, q<0.0001	-0.063 (-0.29 - 0.16), p=0.59, q=0.59
**History of ME/CFS/Fibromyalgia/Chronic pain**	3.65 (0.65 - 6.66), p=0.018, q=0.044	2.42 (-0.13 - 4.98), p=0.064, q=0.11	10.64 (4.77 - 16.50), p=0.00043, q=0.0022	8.25 (-1.09 - 17.59), p=0.084, q=0.11	-0.21 (-0.60 - 0.18), p=0.29, q=0.29
**Recovery cluster**	9.11 (7.44 – 10.79), p<0.0001, q<0.0001	6.68 (5.18 – 8.17), p<0.0001, q<0.0001	17.06 (13.83 – 20.28), p<0.0001, q<0.0001	24.01 (18.12 – 29.91), p<0.0001, q<0.0001	-0.27 (-0.60 – 0.063), p=0.12, q=0.12
**Biocognitive profile (D-dimer)**	1.53 (0.68 – 2.38), p=0.00065, q=0.0016	0.96 (0.28 – 1.65), p=0.0071, q=0.0089	3.57 (2.03 – 5.11), p<0.0001, q<0.0001	4.35 (1.75 – 6.94), p=0.0014, q=0.0023	0.0033 (-0.12 – 0.12), p=0.96, q=0.96
**Biocognitive profile (Fibrinogen)**	0.34 (-0.64 – 1.32), p=0.50, q=0.62	0.87 (0.12 – 1.62), p=0.026, q=0.13	0.85 (-0.98 – 2.68), p=0.37, q=0.61	2.41 (-0.52 – 5.33), p=0.11, q=0.28	-0.025 (-0.15 – 0.10), p=0.70, q=0.70

Table S6 – Prediction of individual symptoms at 2-3 years by individual symptoms at 6 months. For fatigue, the inverse scale (i.e. 52-FACIT) is used so that higher scores represent more fatigue.

Predictor (at 6 months)	Outcome (at 2-3 years)	Adjustment (at 6 months)	beta (95% CI)	p	Adjusted R^2
Depression	Depression	-	0.81 (0.67 - 0.96)	<0.0001	49.21
Depression	Anxiety	Anxiety	0.021 (-0.19 - 0.24)	0.85	0.029
Depression	Fatigue	Fatigue	0.84 (0.50 - 1.17)	<0.0001	16.8
Depression	Subjective cognitive deficit (CCI-20)	Subjective cognitive deficit (C-PSQ)	1.02 (0.44 - 1.61)	0.00083	10.24
Depression	Objective cognitive deficit (Cognitron)	Objective cognitive deficit (MoCA)	-0.0087 (-0.04 - 0.023)	0.59	0.39
Anxiety	Depression	Depression	0.66 (0.37 - 0.94)	<0.0001	14.12
Anxiety	Anxiety	-	0.78 (0.65 - 0.92)	<0.0001	51.86
Anxiety	Fatigue	Fatigue	1.03 (0.69 - 1.37)	<0.0001	22.96
Anxiety	Subjective cognitive deficit (CCI-20)	Subjective cognitive deficit (C-PSQ)	1.50 (0.92 - 2.09)	<0.0001	19.41
Anxiety	Objective cognitive deficit (Cognitron)	Objective cognitive deficit (MoCA)	-0.026 (-0.062 - 0.011)	0.17	2.44
Fatigue	Depression	Depression	0.05 (-0.036 - 0.14)	0.26	1.09
Fatigue	Anxiety	Anxiety	0.029 (-0.034 - 0.093)	0.37	0.69
Fatigue	Fatigue	-	0.65 (0.51 - 0.79)	<0.0001	40.9
Fatigue	Subjective cognitive deficit (CCI-20)	Subjective cognitive deficit (C-PSQ)	0.37 (0.092 - 0.64)	0.01	6.47
Fatigue	Objective cognitive deficit (Cognitron)	Objective cognitive deficit (MoCA)	-0.012 (-0.028 - 0.0032)	0.13	3.14
Subjective cognitive deficit (C-PSQ)	Depression	Depression	0.68 (0.16 - 1.21)	0.013	5.83
Subjective cognitive deficit (C-PSQ)	Anxiety	Anxiety	0.41 (-0.01 - 0.82)	0.059	3.39
Subjective cognitive deficit (C-PSQ)	Fatigue	Fatigue	1.66 (0.64 - 2.67)	0.0018	9.38
Subjective cognitive deficit (C-PSQ)	Subjective cognitive deficit (CCI-20)	-	7.64 (6.41 - 8.87)	<0.0001	57.28
Subjective cognitive deficit (C-PSQ)	Objective cognitive deficit (Cognitron)	Objective cognitive deficit (MoCA)	-0.045 (-0.13 - 0.036)	0.28	1.76
Objective cognitive deficit (MoCA)	Depression	Depression	-0.15 (-0.55 - 0.24)	0.45	0.54
Objective cognitive deficit (MoCA)	Anxiety	Anxiety	-0.072 (-0.38 - 0.24)	0.65	0.19
Objective cognitive deficit (MoCA)	Fatigue	Fatigue	0.17 (-0.68 - 1.01)	0.7	0.14
Objective cognitive deficit (MoCA)	Subjective cognitive deficit (CCI-20)	Subjective cognitive deficit (C-PSQ)	-0.79 (-2.09 - 0.51)	0.24	1.53
Objective cognitive deficit (MoCA)	Objective cognitive deficit (Cognitron)	-	0.13 (0.048 - 0.22)	0.0028	10.64

Table S7 – Association between different symptoms at 2-3 years and occupation change (compared to before COVID-19) at 2-3 years. Odds ratios (OR) are provided for a one point change in the corresponding scales (i.e. 1-point change in PHQ-9, GAD-7, 52-FACIT, CCI-20 and a 1-SD decrease in overall cognitive score).

	**OR** (95% CI)	**p-value**	**OR** (sparse)	**RR** (95% CI)	**RR** (sparse)
**Depression**	1.24 (0.98–1.58)	0.075	1	1.15 (0.98–1.36)	1
**Anxiety**	1.18 (0.93–1.50)	0.17	1	1.11 (0.95–1.31)	1
**Fatigue**	1.31 (1.03–1.69)	0.031	1	1.22 (1.02–1.46)	1
**Subjective cognitive decline**	1.54 (1.21–1.98)	0.00051	1.35	1.32 (1.14–1.56)	1.21
**Objective cognitive deficits**	1.51 (1.04–2.22)	0.031	1.13	1.34 (1.07–1.63)	1.08

Table S8 – Association between different cognitive domains at 2-3 years and occupation change at 2-3 years. Each odds ratio (OR) is reported for a 1-SD decrease in the cognitive score for the corresponding task. The first 4 columns refer to results for the univariable regressions while the last column refers to the multivariable sparse (Lasso) regression.

	**OR**	**95% CI** lower bound	**95% CI** upper bound	**p**	**OR** sparse
**2D Manipulation**	1.114	0.934	1.327	0.23	1.000
**Object Memory (immediate)**	1.084	0.890	1.314	0.42	1.000
**Spatial Span**	1.050	0.884	1.238	0.56	1.000
**Object Memory (delayed)**	1.064	0.879	1.284	0.52	1.000
**Simple reaction speed**	1.340	1.165	1.554	<0.0001	1.206
**Cognitive Control**	1.400	1.112	1.775	0.0047	1.272
**Spatial Planning**	0.994	0.802	1.232	0.96	1.000
**Verbal Analogies**	0.989	0.801	1.223	0.92	1.000

Table S9 – Association between different items of the subjective cognitive decline scale (CCI-20) measured at 2-3 years and occupation change at 2-3 years. Each odds ratio (OR) is reported for a 1-point increase in that item. The first 4 columns refer to results for the univariable regressions while the last column refers to the multivariable sparse (Lasso) regression.

	**OR**	**95% CI** lower bound	**95% CI** upper bound	**p**	**OR** sparse
**Item 1**	1.544	1.201	2.003	0.00085	1.007
**Item 2**	1.371	1.077	1.754	0.011	1.000
**Item 3**	1.427	1.117	1.836	0.0049	1.000
**Item 4**	1.527	1.199	1.959	0.00070	1.000
**Item 5**	1.341	1.061	1.698	0.014	1.000
**Item 6**	1.590	1.241	2.052	0.00029	1.011
**Item 7**	1.311	1.035	1.663	0.025	1.000
**Item 8**	1.628	1.276	2.090	0.00010	1.143
**Item 9**	1.330	1.059	1.669	0.014	1.000
**Item 10**	1.451	1.135	1.865	0.0032	1.000
**Item 11**	1.473	1.150	1.897	0.0023	1.000
**Item 12**	1.281	1.013	1.619	0.038	1.000
**Item 13**	1.263	0.994	1.606	0.056	1.000
**Item 14**	1.347	1.063	1.710	0.014	1.000
**Item 15**	1.606	1.263	2.052	0.00012	1.131
**Item 16**	1.472	1.166	1.862	0.0012	1.000
**Item 17**	1.321	1.046	1.669	0.019	1.000
**Item 18**	1.293	1.022	1.637	0.032	1.000
**Item 19**	1.342	1.061	1.695	0.014	1.000
**Item 20**	1.590	1.243	2.048	0.00026	1.048

Table S10 – Result of the Breusch–Pagan test for heteroscedasticity of the models predicting outcomes. Both the p-value for the test of the model with the predictor (‘full’) and the test with only the covariates (‘covariates’) are provided.

**Predictor**	**Outcome**	**p-value (full)**	**p-value (covariates)**
WHO	Cognitron	0.061	0.024
WHO	CCI-20	0.14	0.065
WHO	FACIT	0.29	0.29
WHO	GAD-7	0.081	0.2
WHO	PHQ-9	0.22	0.34
NEWS	Cognitron	0.084	0.056
NEWS	CCI-20	0.056	0.026
NEWS	FACIT	0.39	0.4
NEWS	GAD-7	0.15	0.1
NEWS	PHQ-9	0.32	0.2
Duration of admission	Cognitron	0.026	0.019
Duration of admission	CCI-20	0.081	0.04
Duration of admission	FACIT	0.18	0.21
Duration of admission	GAD-7	0.036	0.11
Duration of admission	PHQ-9	0.14	0.21
ICU	Cognitron	0.16	0.086
ICU	CCI-20	0.36	0.19
ICU	FACIT	0.48	0.23
ICU	GAD-7	0.98	0.89
ICU	PHQ-9	0.93	0.88
PE	Cognitron	0.13	0.077
PE	CCI-20	0.3	0.17
PE	FACIT	0.19	0.096
PE	GAD-7	0.26	0.46
PE	PHQ-9	0.21	0.35
Delirium	Cognitron	0.16	0.11
Delirium	CCI-20	0.26	0.15
Delirium	FACIT	0.29	0.25
Delirium	GAD-7	0.3	0.23
Delirium	PHQ-9	0.081	0.11
Moderate to severe cluster	Cognitron	0.27	0.16
Moderate to severe cluster	CCI-20	0.12	0.55
Moderate to severe cluster	FACIT	0.0039	0.68
Moderate to severe cluster	GAD-7	0.14	0.79
Moderate to severe cluster	PHQ-9	0.12	0.95
Very severe cluster	Cognitron	0.22	0.1
Very severe cluster	CCI-20	0.29	0.22
Very severe cluster	FACIT	0.0091	0.28
Very severe cluster	GAD-7	0.21	0.92

Very severe cluster	PHQ-9	0.32	0.96
Biocognitive profile (Fibrinogen)	Cognitron	0.14	0.11
Biocognitive profile (Fibrinogen)	CCI-20	0.16	0.29
Biocognitive profile (Fibrinogen)	FACIT	0.41	0.5
Biocognitive profile (Fibrinogen)	GAD-7	0.58	0.93
Biocognitive profile (Fibrinogen)	PHQ-9	0.74	0.74
Biocognitive profile (D-dimer)	Cognitron	0.17	0.11
Biocognitive profile (D-dimer)	CCI-20	0.47	0.29
Biocognitive profile (D-dimer)	FACIT	0.62	0.5
Biocognitive profile (D-dimer)	GAD-7	0.98	0.93
Biocognitive profile (D-dimer)	PHQ-9	0.67	0.74
History of psychiatric/neurological comorbidity	Cognitron	0.038	0.019
History of psychiatric/neurological comorbidity	CCI-20	0.034	0.06
History of psychiatric/neurological comorbidity	FACIT	0.24	0.27
History of psychiatric/neurological comorbidity	GAD-7	0.066	0.13
History of psychiatric/neurological comorbidity	PHQ-9	0.032	0.26
History of ME/CFS/Fibromyalgia/Chronic fatigue	Cognitron	0.04	0.019
History of ME/CFS/Fibromyalgia/Chronic fatigue	CCI-20	0.079	0.06
History of ME/CFS/Fibromyalgia/Chronic fatigue	FACIT	0.37	0.27
History of ME/CFS/Fibromyalgia/Chronic fatigue	GAD-7	0.22	0.13
History of ME/CFS/Fibromyalgia/Chronic fatigue	PHQ-9	0.37	0.26

References

1 Evans RA, McAuley H, Harrison EM, *et al.* Physical, cognitive, and mental health impacts of COVID-19 after hospitalisation (PHOSP-COVID): a UK multicentre, prospective cohort study. *Lancet Respir Med* 2021; **9**: 1275–87.

2 Cohort Profile: Post-Hospitalisation COVID-19 (PHOSP-COVID) study. *International Journal of Epidemiology*.

3 Taquet M, Skorniewska Z, Hampshire A, *et al.* Acute blood biomarker profiles predict cognitive deficits 6 and 12 months after COVID-19 hospitalization. *Nat Med* 2023; published online Aug 31. DOI:10.1038/s41591-023-02525-y.

4 PHOSP-COVID Collaborative Group. Clinical characteristics with inflammation profiling of long COVID and association with 1-year recovery following hospitalisation in the UK: a prospective observational study. *Lancet Respir Med* 2022; **10**: 761–75.

5 Taquet, Maxime, Zuzanna Skorniewska, Henrik Zetterberg, John R. Geddes, Catherine J. Mummery, James D. Chalmers, Ling-Pei Ho et al. Post-acute COVID-19 neuropsychiatric symptoms are not associated with ongoing nervous system injury. *Brain Communications* 2024; **6**: fcad357.

6 Rattanabannakit C, Risacher SL, Gao S, *et al.* The Cognitive Change Index as a measure of self and informant perception of cognitive decline: Relation to neuropsychological tests. *J Alzheimers Dis* 2016; **51**: 1145–55.

7 Hampshire A, Trender W, Chamberlain SR, *et al.* Cognitive deficits in people who have recovered from COVID-19. *EClinicalMedicine* 2021; **39**: 101044.

8 Montan I, Löwe B, Cella D, Mehnert A, Hinz A. General population norms for the Functional Assessment of Chronic Illness Therapy (FACIT)-Fatigue Scale. *Value Health* 2018; **21**: 1313–21.

9 Risacher SL, Kim S, Nho K, *et al.* APOE effect on Alzheimer's disease biomarkers in older adults with significant memory concern. *Alzheimers Dement* 2015; **11**: 1417–29.

10 Risacher SL, Tallman EF, West JD, *et al.* Olfactory identification in subjective cognitive decline and mild cognitive impairment: Association with tau but not amyloid positron emission tomography. *Alzheimers Dement (Amst)* 2017; **9**: 57–66.

11 Agresti A, Coull BA. Approximate is better than "exact" for interval estimation of binomial proportions. *Am Stat* 1998; **52**: 119–26.
